# Hyperfunctional complement C3 promotes C5-dependent atypical hemolytic uremic syndrome in mice

**DOI:** 10.1172/JCI99296

**Published:** 2019-02-04

**Authors:** Kate Smith-Jackson, Yi Yang, Harriet Denton, Isabel Y. Pappworth, Katie Cooke, Paul N. Barlow, John P. Atkinson, M. Kathryn Liszewski, Matthew C. Pickering, David Kavanagh, H. Terence Cook, Kevin J. Marchbank

**Affiliations:** 1 Institute of Cellular Medicine, Newcastle University, Newcastle upon Tyne, United Kingdom.; 2 The National Renal Complement Therapeutics Centre (NRCTC), Newcastle upon Tyne Hospitals NHS Foundation Trust, Newcastle upon Tyne, United Kingdom.; 3 Department of Chemistry, University of Edinburgh, Edinburgh, United Kingdom.; 4 Division of Rheumatology, Washington University in St. Louis, St. Louis, Missouri, USA.; 5 Department of Medicine, Imperial College London, London, United Kingdom.

**Keywords:** Immunology, Nephrology, Chronic kidney disease, Complement, Innate immunity

## Abstract

Atypical hemolytic uremic syndrome (aHUS) is frequently associated in humans with loss-of-function mutations in complement-regulating proteins or gain-of-function mutations in complement-activating proteins. Thus, aHUS provides an archetypal complement-mediated disease with which to model new therapeutic strategies and treatments. Herein, we show that, when transferred to mice, an aHUS-associated gain-of-function change (D1115N) to the complement-activation protein C3 results in aHUS. Homozygous C3 p.D1115N (C3KI) mice developed spontaneous chronic thrombotic microangiopathy together with hematuria, thrombocytopenia, elevated creatinine, and evidence of hemolysis. Mice with active disease had reduced plasma C3 with C3 fragment and C9 deposition within the kidney. Therapeutic blockade or genetic deletion of C5, a protein downstream of C3 in the complement cascade, protected homozygous C3KI mice from thrombotic microangiopathy and aHUS. Thus, our data provide in vivo modeling evidence that gain-of-function changes in complement C3 drive aHUS. They also show that long-term C5 deficiency is not accompanied by development of other renal complications (such as C3 glomerulopathy) despite sustained dysregulation of C3. Our results suggest that this preclinical model will allow testing of novel complement inhibitors with the aim of developing precisely targeted therapeutics that could have application in many complement-mediated diseases.

## Graphical abstract

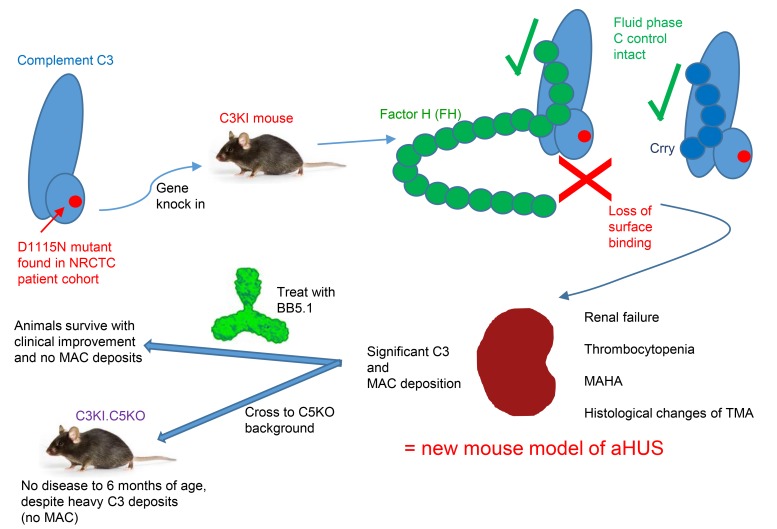

## Introduction

Our understanding and treatment of complement-mediated diseases has grown exponentially over the last 20 years. We now understand that complement plays an integral role in many autoimmune- and inflammation-based diseases or conditions (1). The identification of hyperactive complement as the primary driver in atypical hemolytic uremic syndrome (aHUS) (2) and its largely successful treatment using eculizumab (3, 4) has revealed the potential of anti-complement therapy in the treatment of many diseases, such as age-related macular degeneration (AMD), autoimmune disorders, and neurodegenerative conditions (5). However, eculizumab is not a panacea in AMD (6) or rheumatic diseases (7) or even in aHUS (8). Thus, there remains a need for better models of complement-mediated diseases to trial drugs and dissect the molecular pathways that are common among complement-mediated/associated diseases.

The complement system, consisting of more than 50 proteins, is integral to defense against pathogens and for maintenance of host homeostasis. It is considered the backbone of innate immunity, as it is critical in microbial killing, apoptotic cell clearance, and immune complex handling (9). Key complement proteins are activated in a stepwise manner through an enzymatic activation cascade that is regulated by an array of modulators (10). Activation can be achieved through one or more of the classical, lectin, and alternative pathways, all of which culminate in the cleavage of C3. Cleavage of C3 (185 kDa) into C3b (177 kDa) and C3a (8 kDa) is the central activating step of the complement cascade (11). C3 cleavage leads to deposition of C3b onto the surface of microbes (opsonization), release of the proinflammatory anaphylactic products C3a and C5a, and initiation of the terminal pathway, leading to the formation of the membrane attack complex (MAC) C5b-9 and cell lysis (12). Given the intricate nature of the complement cascade, many soluble and membrane-associated proteins function to regulate C3 activation (13). Together these protect healthy host cells and tissues from unwanted complement activation, while unprotected surfaces rapidly become coated in C3b molecules (1). Abnormalities in the complement system, derived by genetic or acquired means, are associated with a variety of pathologies (14).

aHUS is a renal disease that encompasses the clinical triad of microangiopathic hemolytic anaemia, thrombocytopenia, and acute renal failure (15). Dysregulation of the alternative pathway of the complement system at the cell or extracellular matrix surface is a major factor in susceptibility to aHUS (16). In particular, an imbalance between the activators and regulators of the alternative pathway results in complement-mediated damage to the endothelium (17). Approximately 60% of individuals with aHUS have at-risk mutations in their complement genes (4). Mutations occur either in the genes encoding complement-regulating proteins such as factor H (FH), factor I (FI), and membrane cofactor protein (MCP) or in genes encoding complement-activation proteins (C3 and factor B [FB]) (18). In general, it has been observed that disease-linked mutations in regulators result in a loss of function, while disease-linked mutations in activators result in a gain of function (19). The most frequently mutated gene in aHUS patients is *CFH* (the gene encoding FH), occurring in 25% of sporadic cases, followed by mutations in *C3* (4%–11% of cases), in *CFI* (5%–10%), in *MCP* (5%–9%), and in *FB* (<4%) (20).

The nature of the genetic complement abnormality has a significant impact on the clinical phenotype. Those with mutations in *MCP* have the best prognosis (only 6% reach end-stage renal failure [ESRF]), while mutations in *CFH* have historically been associated with the worst prognosis, including earlier disease onset and higher risk of relapse (20). *CFH* mutations typically cluster in exons 22 and 23 of *CFH*, which encode the C-terminal short consensus repeats (SCRs; also referred to as complement control protein domains [CCPs]) 19–20 of FH (21, 22). This region is important for regulating C3b amplification on host cell and tissue surfaces, while the N-terminal region SCRs 1–4 are important for regulatory activity both in the fluid phase and on surfaces (21, 23). The functional significance of the SCRs 19–20 was elegantly illustrated through transgenic expression of an FH truncation protein that lacked the C-terminal 5 SCRs backcrossed onto FH-knockout mice (24). These mice spontaneously developed features mirroring aHUS in humans. This observation indicated that regulation in the fluid phase (preventing futile depletion of C3 in plasma) combined with dysregulation at the cell surface was pathogenic in aHUS. Further work confirmed the damage caused by downstream events in the complement cascade, since the phenotype could be rescued by breeding onto a *C5*-deficient background (25). We have known for some time that gain-of-function C3 mutations, clustering in the FH-binding interface of C3b, are associated with aHUS (26). Interestingly, mutations in *C3* have varied outcomes, with 63%–67% of adult patients progressing rapidly to ESRF and some *C3* mutations (including D1115N) resulting in chronic progression (27, 28), a renal prognosis similar to that seen with many *CFH* mutations (15). As SCRs 19–20 of FH bind to the thioester domain (TED) of C3b, it was expected that in vitro studies would confirm that recombinant human C3 proteins with mutations located in the TED (mature C3 p.D1093N, p.C1136W, p.Q1139K, p.A1072V) were defective in binding to FH (26, 29). This reduced binding affinity was proposed to cause reduced proteolytic inactivation and therefore enhanced complement activation in vivo. To date, however, no in vivo studies have examined C3 gain-of-function aHUS mutations. Consequently, a direct demonstration that these mutations cause aHUS has been lacking.

The historical work in animal models, coupled with the successful use of C5 inhibitors in aHUS patients, lays the foundation for our current understanding of the pathogenesis of aHUS (30). However, what is not yet known is the effects of long-term C5 inhibition in the presence of continued dysregulation of the alternative pathway (31). In theory, with time, patients on C5-inhibiting therapy could convert their phenotype from aHUS to a C3-mediated glomerular disease (e.g., complement 3 glomerulopathy [C3G]; refs. 32, 33) as opposed to a C5/terminal pathway–mediated disease. Lifelong C5 inhibition poses a significant immunosuppressive burden on the patient (34), particularly patients who have renal transplants and are already on triple immunosuppressive therapy (35). While previous (24) and existing mouse models based on FH functional deficit (36, 37) display some of the characteristic phenotypes/pathologies of aHUS, reflecting potential subsets of the disease, they do not entirely reproduce the clinical observations, i.e., disease induction required either nephrotoxic serum (36) or was driven by excessive coagulation/thrombosis (37).

Herein, we describe the transfer of human C3 gain-of-function aHUS-associated mutations to murine C3. Following in vitro characterization of these murine C3 mutants, we introduced a point mutation in murine *C3* (p.D1115N) in conditional knockin mice. C57BL/6 mice homozygous for C3 Asn1115 (C3KI) exhibit a spontaneous aggressive renal phenotype that is identical to aHUS as described in humans. Ex vivo analysis confirmed that failure to bind FH could explain the pathogenicity of this point mutant. C5 blockade protected C3KI mice from disease, and through long-term studies of C5 genetic deletion, we have shown that while increased C3 turnover continues through dysregulation of the alternative pathway, this does not evolve into a C3 glomerulopathy. Given that our model robustly recapitulates the clinical phenotype in humans, this allows us to translate our long-term studies in mice to humans — providing insight into the effects of chronic terminal pathway inhibition on the alternative pathway. This C3 gain-of-function mouse model of aHUS thus provides an opportunity for anti-complement drug testing.

## Results

### Natural history of a familial C3 Asn1115 mutation discovered in the NRCTC aHUS cohort.

**Figure 1:**
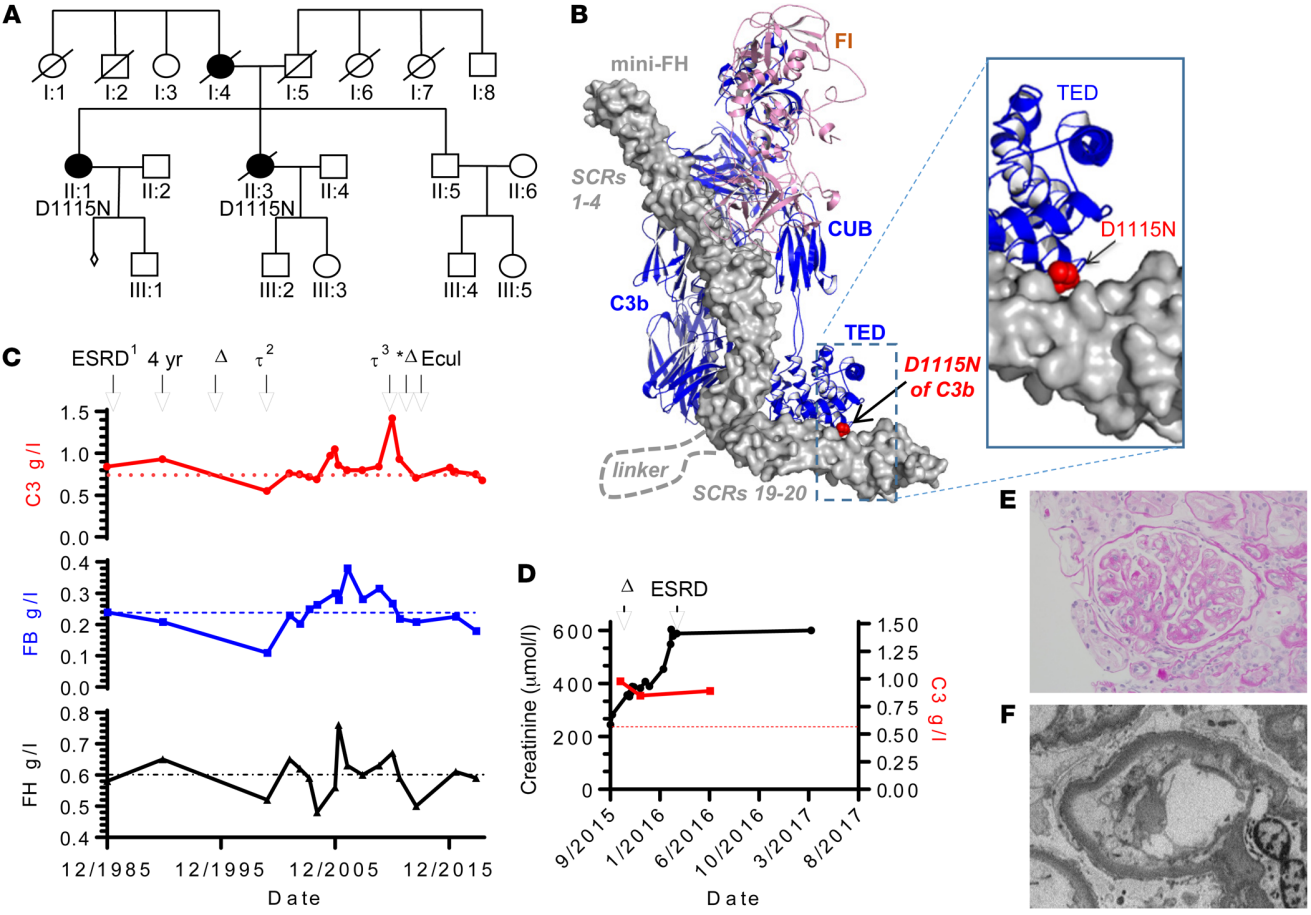
The NRCTC family heterozygous for C3 Asn1115 mutation. (**A**) Family pedigree showing affected individuals. (**B**) Location of the C3 mutation (D1115N) within the activated C3b fragment. The structure shown is the complex of human C3b, human FI, and mini-FH (SCRs 1–4 and SCRs 19–20, of human FH, connected by a poly-Gly linker) (5o32.pdb). C3b (blue) and FI (pink) are shown as cartoons, and mini-FH (gray) as a surface. The side chain atoms of C3b residue D1115 are shown as red spheres. CUB, complement C1r/C1s, Uegf, Bmp1. (**C**) Complement profile and renal events of II:1. Where available, C3 levels (top panel) with lower reference range (dotted line); FB levels (middle panel) with lower reference range (dashed line); and FH levels (bottom panel) with upper reference range (black dash-dotted line) are shown over more than 30 years. Associated clinical features are indicated above: ERSD and received first renal allograft, fourth year after transplant; Δ indicates declining graft function as a result of chronic allograft nephropathy; the second and third renal allografts are indicated by τ^2^ and τ^3^, respectively, with asterisk highlighting loss of third renal allograft to recurrent aHUS with increased creatinine and treatment with eculizumab (Ecul). (**D**) Where available, C3 and creatinine levels of individual II:3; C3 level in red (right *y* axis), and renal function in black; Δ indicates chronic TMA on biopsy followed by ESRD as indicated. (**E**) Representative H&E staining of II:3 showing double contouring (a feature of TMA); original magnification, ×40. (**F**) Electron micrograph from II:3 shows a capillary loop with detachment of the endothelium with accumulation of electron lucent material; original magnification, ×8000.

The C3 Asn1115 point mutation has been identified in 3 families, including in the NRCTC/Paris aHUS patient cohort (Figure 1A) (26). The patients are heterozygous for a missense mutation in *C3* (*C3* c.3343 G>A), corresponding to replacement of an aspartic acid with an asparagine at position 1115 of C3 (D1115N, Figure 1B; note this is D1093N in the mature protein) (15, 26, 28). The C3 Asn1115 variant has a substantial loss in binding to both MCP (CD46) and FH (26, 29), which we confirmed in additional surface plasmon resonance (SPR) analysis (Supplemental Figure 1; supplemental material available online with this article; https://doi.org/10.1172/JCI99296DS1). Despite almost complete loss of interaction with MCP, we hypothesized that the loss of interaction of the C-terminal region FH with the TED of this C3b (the cleaved, activated form of C3) variant, as illustrated in Figure 1B, would be important for selective recognition of a self-surface (38), which could be critical for the pathogenicity of the mutation. The chronological clinical parameters of creatinine and C3, FB, and FH of the family carrying the Asn1115 mutation are presented as available (Figure 1, C and D). One patient has a functioning renal transplant (28). This patient received the transplant before the era of preemptive eculizumab for kidney transplantation, and when graft function started to decline, a biopsy confirmed features of a chronic thrombotic microangiopathy (TMA; Figure 1, E and F). In the context of low-grade hemolysis, eculizumab treatment was commenced for recurrent aHUS (Figure 1C). This was successful, with no further deterioration in allograft function to date (albeit chronic damage had already occurred prior to the use of eculizumab). Notably in this patient, FH plasma levels were found to be above the upper limits of the normal range for the majority of the time points analyzed, while FB levels were below the normal range (Figure 1C), providing evidence of increased alternative pathway activation.

### Successful generation and functional testing of recombinant mouse C3 and several disease-associated mutants.

Data generated in vitro with mutant human C3 molecules have suggested that some are resistant to control by FH (26, 29), but there has been no direct in vivo demonstration that mutations in C3, including those identified in the Newcastle patients, cause aHUS. To address this, we sought to develop a new animal model. Initially, we needed to test the feasibility of transferring mutations in human C3 and their functional outcomes to murine C3. A pairwise sequence alignment confirmed that all mutated C3 residues identified in our earlier study (26) and several others identified later (29) were conserved in mouse C3, with the exception of R102 (Supplemental Figure 2A). Indeed, this pattern of residue conservation was noted across rat (P01026), bovine (Q2UVX4), pig (P01025), and guinea pig (P12387) C3 proteins (Supplemental Figure 2B). For ease of purification, we designed a cDNA construct to allow production of recombinant FLAG- and 7xHis-tagged murine C3 (NM_009778.3) in mammalian tissue culture (see Supplemental Methods and Supplemental Figure 3). After sequence confirmation of the recombinant mouse C3 (rmC3) construct, we used site-directed mutagenesis to generate 10 mutant rmC3 clones. These included 923ΔDG rmC3, based on the previous findings of gain of function for this mutant associated with C3 glomerulopathy (Supplemental Figure 2C) (39). The recombinant proteins were purified to homogeneity using FLAG-tag affinity chromatography and examined by SDS-PAGE (Supplemental Figure 4). Using standard fluid-phase cofactor assays, we found evidence that rmC3b (Val1072) and rmC3b (Asn1115), in comparison to WT rmC3b, demonstrated clear resistance to proteolytic inactivation by FI (Supplemental Figure 4). Furthermore, using SPR (Biacore), several of our C3b mutants had reduced binding to “mini” murine FH (mini-FH; a construct containing the 2 key C3b-binding sites, i.e., SCRs 1–5 and SCRs 18–20 of murine FH), with rmC3(Asn1115) having one of the lowest-affinity interactions in this assay (Supplemental Figure 5). This is entirely consistent with loss of an important H-bonding interaction with Tyr1190 of FH as described by Morgan et al. (38). To explore the in vivo consequence of C3 gain-of-function changes, we next generated a conditional knockin mouse (C3KI) based on the Asn1115 change.

### Development of the C3KI model.

**Figure 2:**
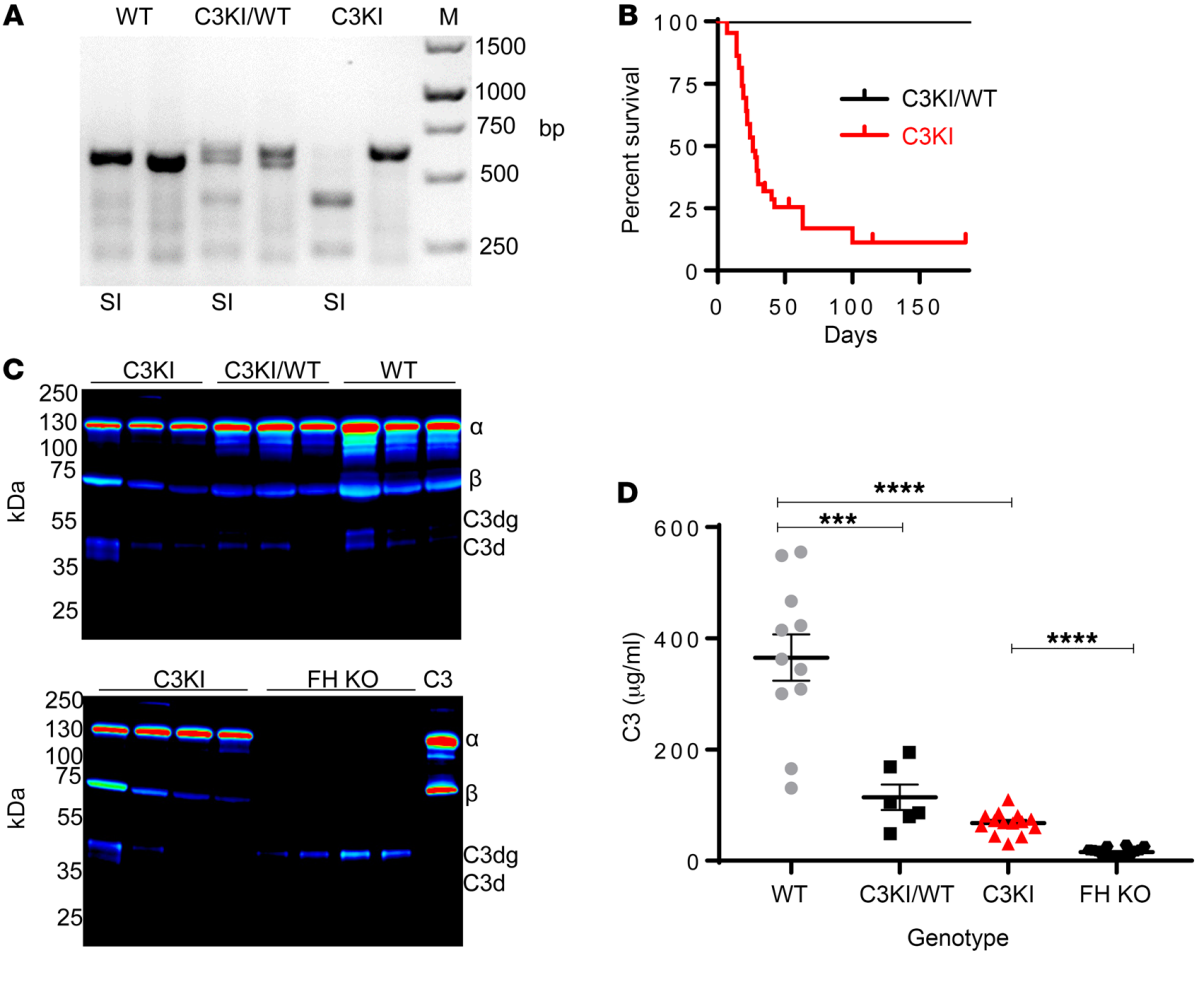
Initial characterization of C3KI mice. (**A**) A representative ethidium bromide–stained PCR gel illustrating genotyping of C3KI mice, with the SalI digest, as appropriate, indicated by SI below the gel. (**B**) Kaplan-Meier survival curve analysis of the first 44 C3KI and C3KI/WT littermates (F_1_–F_3_). Data for 44 WT littermates were identical to those for C3KI/WT. (**C**) Western blots of murine C3 derived from plasma (freshly collected into EDTA and run under reduced conditions) detected by goat anti–mouse C3 (MP Biomedicals). Mouse genotype is indicated above the lanes; each lane represents an individual mouse. Chemiluminescence image were captured using LI-COR Odyssey FC and displayed in pseudo-color. (**D**) Plasma murine C3 levels from 11 WT (gray circles), 6 knockin (KI)/WT (black squares), 15 KI (red triangles), and 16 FH-KO (black hexagons) mice were measured. Absorbance readings at 450 nm were converted to μg/ml using a standard curve based on purified mouse C3; mean ± SEM. ***P < 0.001 and ****P < 0.0001 using unpaired *t* test with Welch’s correction.

Possession of a form of C3b that cannot be recognized by FH is expected to be embryonically lethal (40). Thus, C3KI mice were generated using conditional knockin technologies (Ozgene; see Supplemental Figure 6). When these mice were bred onto constitutive Cre-expressing mice (B6^Tm1Cre/Oz^), heterozygous (C3KI/WT) and homozygous (C3KI) mice were readily identified by genotype PCR (Figure 2A). Subsequent analysis of F_2_ litters showed that proportions of specific genotypes were in line with expectations (20% WT versus 28% C3KI, with no influence on sex; Supplemental Figure 7A) from a normal Mendelian mode of inheritance, indicating that the D1115N mutation in C3 did not affect embryo survival in utero. At weaning (aged 18–21 days), many C3KI mice weighed less than their littermates (Supplemental Figure 7B), and by postpartum day 50 (P50) they exhibited significantly (*P* < 0.0001) higher mortality (Figure 2B). Analysis of plasma from C3KI/WT and C3KI mice showed the presence of intact mutant C3 protein and some evidence of C3 breakdown fragments, i.e., C3d, but not complete loss of intact C3 as observed in FH-deficient mice (Figure 2, C and D). See complete unedited blots in the supplemental material. Similar evidence was noted with respect to FB consumption (Supplemental Figure 7C). We suspect that C3b amplification is less well regulated, leading to an increased rate of conversion of C3 Asn1115 to C3b Asn1115 and low C3 levels (68 ± 5 μg/ml C3KI versus 365± 41 μg/ml for WT). However, the degree of C3 consumption/fragment generation was significantly lower than that seen in the FH-deficient mouse, where very little intact C3 was detectable in isolated plasma (15 ± 3 μg/ml; Figure 2, C and D) (36, 41).

### Characterization of C3KI mice.

**Figure 3:**
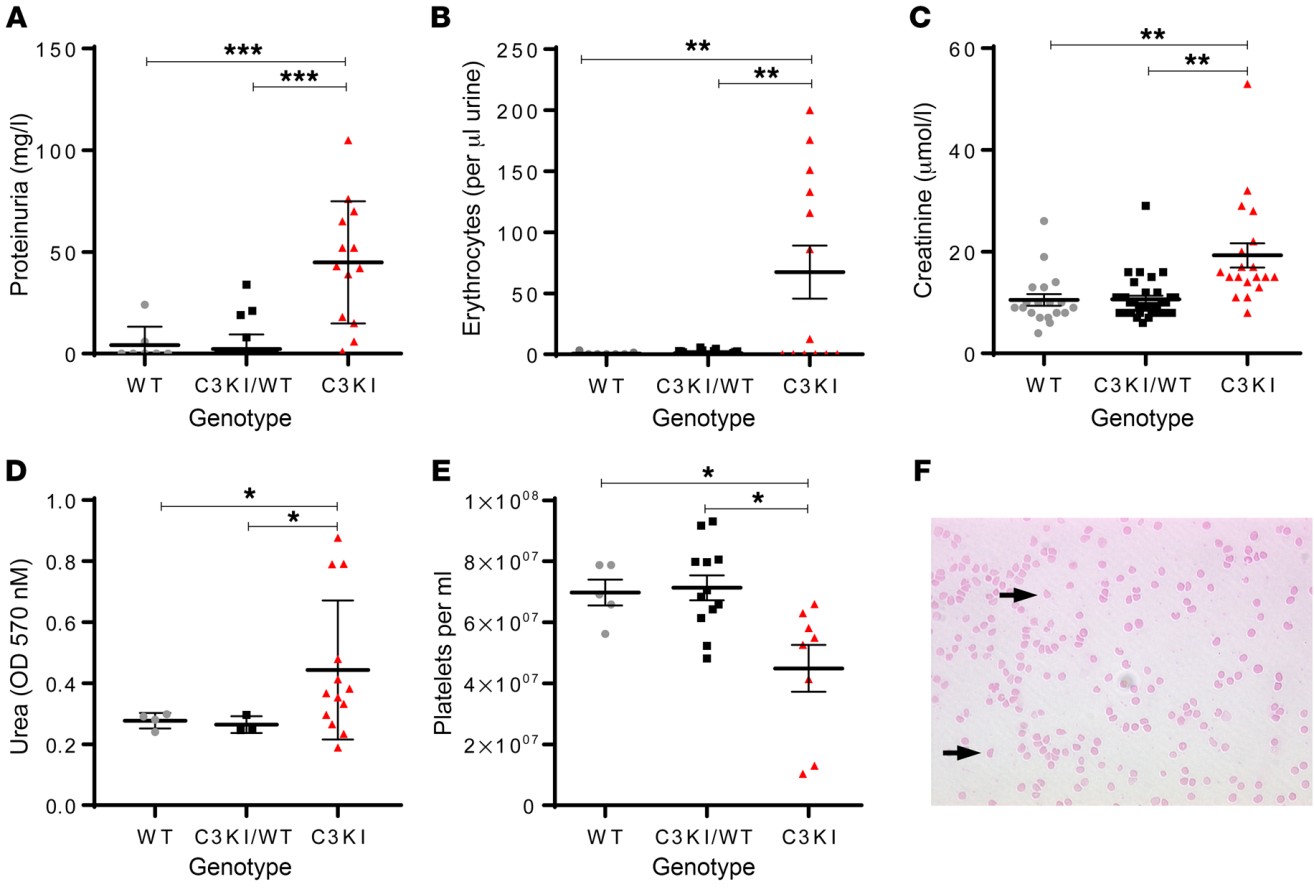
Further characterization of the C3KI mice. Where available, levels of proteinuria (**A**) and hematuria (**B**) were measured by Combistix (Siemens/Bayer) analysis of urine collected during routine handling. Results for 13 C3KI (triangles), 24 C3KI/WT (squares), and 8 WT (circles) mice, P14–P21, are shown. (**C**) Creatinine values obtained from the mouse plasma taken from 19 C3KI, 34 C3KI/WT heterozygous, and 19 WT mice, P14–P28, are shown. (**D**) Urea values obtained from plasma taken from 13 C3KI mice, 4 C3KI/WT, and 4 WT mice. (**E**) Platelet counts of 8 C3KI, 12 C3KI/WT, and 5 WT mice on P21. Absolute numbers were provided by use of cell count beads and flow cytometry. (**F**) A representative (*n* = 8, P21) Giemsa-stained blood film from a C3KI mouse showing schistocytes (indicated by black arrows), consistent with mechanical hemolysis of red blood cells. Original magnification ×40. *P < 0.05, **P < 0.01, ***P < 0.005 using unpaired *t* test with Welch’s correction.

Analysis of survival curves showed clearly that (homozygous) C3KI mice, but not (heterozygous) C3KI/WT mice, had a higher mortality than WT mice. We therefore decided to track C3KI mice from P7. C3KI mice were smaller than littermates at all stages of analysis, and many C3KI mice displayed heavy proteinuria and significant hematuria (Figure 3, A and B). Creatinine levels (reflecting renal impairment) were elevated in the majority of C3KI mice, but were highest in mice in the final stages of disease, as expected (Figure 3C). Additionally, urea levels, another surrogate marker of renal dysfunction, were elevated in the majority of C3KI mice (Figure 3D). Platelet counts were lower in C3KI mice, and very sick animals presented with severe thrombocytopenia (Figure 3E). Blood films from C3KI mice contained schistocytes, indicating fragmentation of red blood cells (Figure 3F). The presence of schistocytes coupled with the observation of thrombocytopenia are consistent with microangiopathic hemolytic anemia, a salient feature of aHUS.

### TMA in C3KI mice.

**Figure 4:**
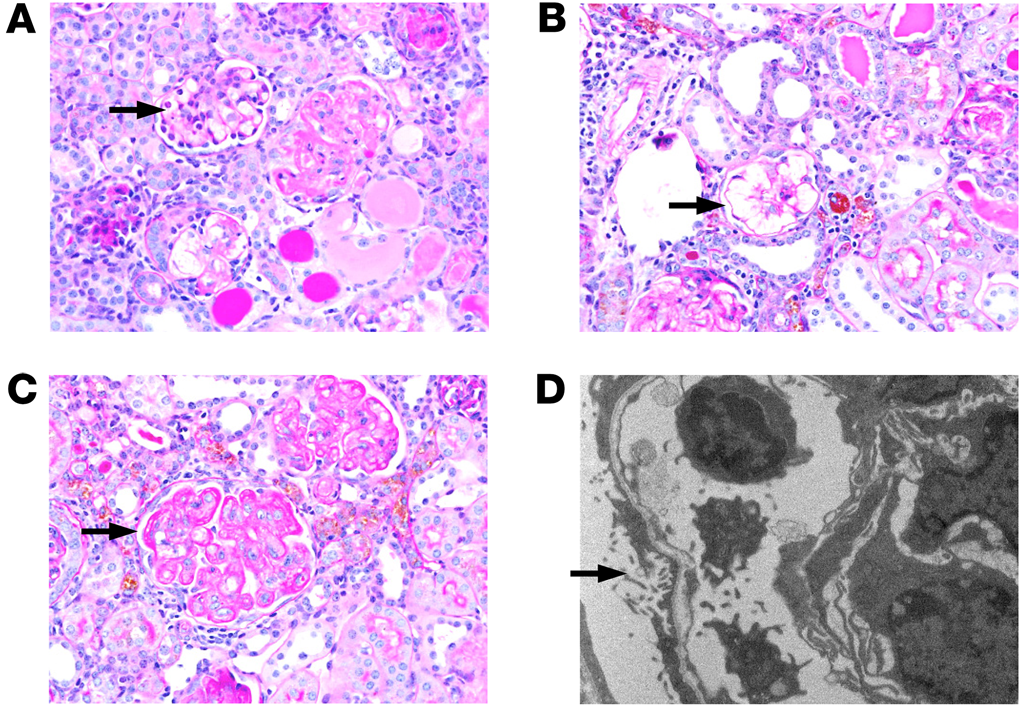
TMA in C3KI mice. PAS-stained sections from C3KI mice aged 14–28 days. Images are representative of the 8 C3KI mice examined. Arrows highlight key examples of the following: (**A**) Mesangiolysis in the glomerulus. (**B**) Microaneurysms of the glomerular capillaries following dissolution of the matrix after the mesangiolysis. (**C**) Double contouring of glomerular capillary walls, a key feature of chronic TMA. Original magnification in **A**–**C**, ×40. (**D**) Electron microscopy (original magnification, ×7900) showing subendothelial lucency, which is consistent with ischemic conditions precipitated by the TMA from a day 7 C3KI mouse.

C3KI mice had renal disease, as evidenced by proteinuria, hematuria and elevated creatinine, and a proportion reached ESRF, requiring euthanasia. Periodic acid–Schiff (PAS) staining of kidney sections, from C3KI mice ranging from 14 to 28 days old, was consistent with histological features typical of a chronic TMA, including mesangiolysis (Figure 4A), followed by microaneurysm formation resulting from the dissolution of the mesangial matrix (Figure 4B) and double contouring of the glomerular basement membrane (Figure 4C). Electron microscopy confirmed endothelial injury with loss of fenestrations and subendothelial lucency (Figure 4D). Fibrin deposition within glomeruli was observed in C3KI mice, and occasionally, intravascular thromboses were identified (Supplemental Figure 7, D–I). Interestingly, we did not see any histological evidence of a TMA in the C3KI/WT mice (Supplemental Figure 8, A–C), MAHA (Supplemental Figure 8D), or kidney disease even after a year (Supplemental Figure 8E).

### Complement dysregulation of C3KI.

**Figure 5:**
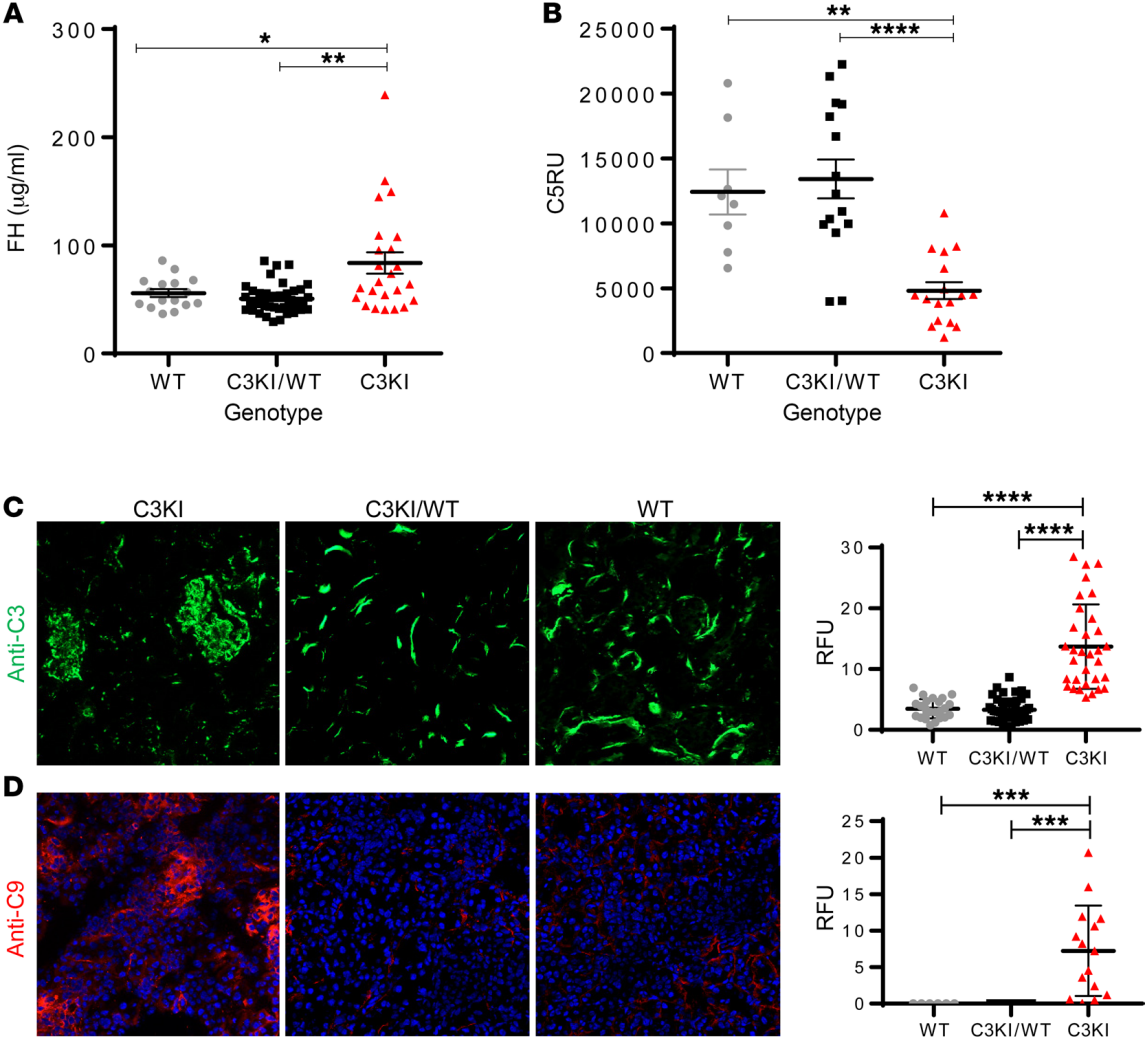
Complement dysregulation in C3KI mice. (**A**) Complement FH levels in mouse plasma isolated from 19 C3KI mice (triangles), 34 C3KI/WT (squares), and 19 WT (circle) mice, P14–28, were established using an in-house sandwich ELISA described in Methods. (**B**) C5 levels in mouse plasma isolated from 17 C3KI, 15 C3KI/WT, and 8 WT mice on P14–P28 were established using an in-house sandwich ELISA. Relative units were generated via a standard curve based on WT control serum. (**C**) Immunofluorescence of glomerular C3 deposition in C3KI, C3KI/WT, and WT kidneys (representative of *n* = 10 in each group). Images were taken on a Zeiss Axio Imager II. Original magnification, ×20. Densitometry analysis is shown to the right of the panels; relative fluorescence units from >25 image captures are indicated. (**D**) Glomerular C9 deposition in C3KI in comparison to background staining of WT. Original magnification ×20; >6 image captures shown in densitometry. *P < 0.05, **P < 0.005, ***P < 0.001, ****P < 0.0001 using unpaired *t* test with Welch’s correction.

Having identified an aggressive renal disease in the C3KI mice, coupled with the histological analysis showing a TMA (in keeping with aHUS), we investigated the complement profile of the C3KI mice. Interestingly, plasma FH levels in C3KI mice were elevated (Figure 5A). This is consistent with a response to higher levels of complement activation or regulation (37). Additionally, plasma C5 levels were lower in C3KI mice (Figure 5B). Significant C3 fragment deposition was identified using immunofluorescence staining (Figure 5C). A granular staining pattern, largely confined to the mesangium and capillary walls of the glomerulus, was clearly evident in the C3KI mice. This was distinct from the C3KI/WT and WT littermates, in which tubulointerstitial staining was observed (which is normal in murine kidneys; refs. 24, 37). Detection of C9 (Figure 5D) in the glomeruli of C3KI mice, by immunofluorescence staining, suggested that terminal pathway activation and MAC deposition contribute to disease in the C3KI mice, as also happens during aHUS in humans. No glomerular C9 deposition was found in the kidney of C3KI/WT or WT mice (Figure 5D).

### Molecular basis for complement dysregulation.

**Figure 6:**
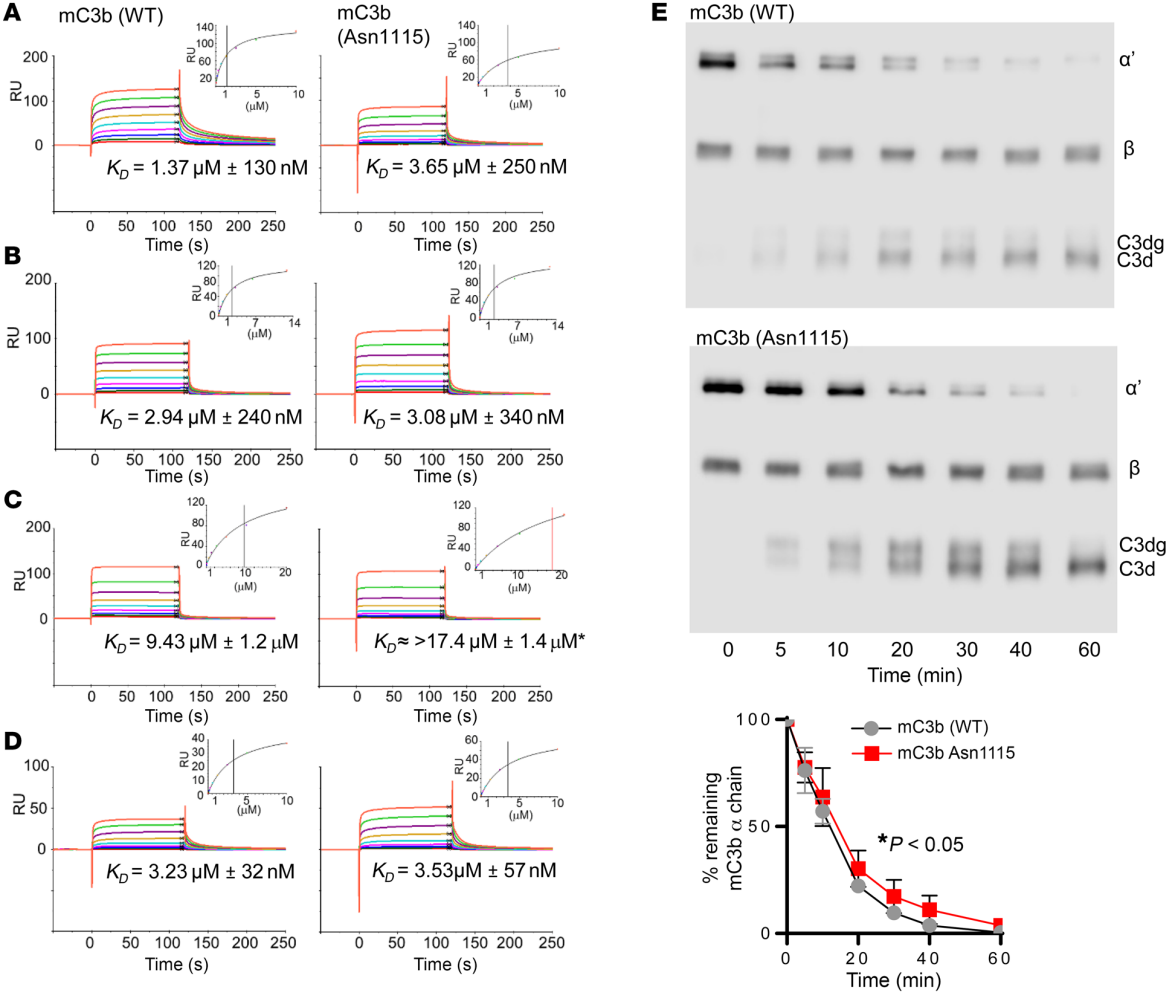
Molecular basis of complement dysregulation in C3KI mice. SPR analysis using mouse C3b derived from WT or C3KI mouse plasma. Mouse C3b was amine coupled to a CM5 biosensor chip (930 ± 10 RU). Doubly diluted concentration series of (**A**) purified murine FH (0 to 10 μM), (**B**) recombinant murine FH SCRs 1–5 (0 to 14.6 μM), (**C**) recombinant human FH SCRs 19-20 (0 to 20 μM), or (**D**) recombinant murine Crry SCRs 1–5 (0 to 10 μM) were flowed across the chip surface. The equilibrium dissociation constant *K_D_* was calculated using a steady-state model in the Biacore evaluation package, indicated as the black vertical line. *At the concentration range assayed here, human FH19–20 was not able to achieve saturated binding on the C3b Asn1115 surface; thus, an underestimated *K_D_* is indicated by the red vertical line. (**E**) Representative gels showing plasma-purified murine C3, as indicated, incubated with human FI and FH in a fluid phase cofactor activity assay, with densitometry analysis (Image Studio version 5.2) of intact α chain shown below; shown is the average of 3 experiments ± SEM with unpaired *t* test.

Based on our work using rmC3 (Supplemental Figures 4 and 5), we expected C3 isolated from the blood of C3KI mice to have lower affinity, compared with WT murine C3, for FH. To confirm this, we isolated and purified C3 Asn1115 protein from knockin mice to homogeneity, and purified WT C3 as well as full-length FH from C57BL/6 mice. In addition, we generated recombinant mouse FH1–5, mouse Crry, and human FH19–20 (see Supplemental Methods for details). Using SPR, we measured the strength of the interaction between WT murine C3b or murine C3b Asn1115 (generated by incubation with human FB and FD; see Methods; 930 ± 10 RU amine coupled to a CM5 chip) and regulatory proteins or protein fragments. Full-length mouse serum–derived FH bound to WT C3 with an affinity (*K_D_*) of 1.4 ± 0.13 μM, while it bound C3b Asn1115 with a *K_D_* of 3.7 ± 0.25 μM (Figure 6A). Recombinant mouse FH1–5 bound to both WT C3b and C3b Asn1115 with essentially equivalent affinity (2.9 ± 0.24 μM and 3.1 ± 0.34 μM, respectively; Figure 6B). These values are broadly similar to the affinity of full-length mouse FH binding to C3b Asn1115, and hence, taken together, these SPR data suggest the almost complete loss of binding between mutant C3 and the C-terminus of FH. We confirmed this using SPR to measure the binding between human FH19–20 (42, 43) (the analyte) and various versions of mouse C3b. FH19–20 bound WT C3b with a *K_D_* of 9.4 ± 1.2 μM (43), whereas it bound immobilized C3b Asn1115 with a much weaker *K_D_* (saturation was not reached) of >17.4 (Figure 6C). Finally, as Crry is the major cell surface regulator of complement in the mouse, we also evaluated its binding to WT and mutant C3b and found that recombinant mouse Crry bound to WT C3b and C3b Asn1115 with essentially equivalent affinity (3.2 ± 0.03 μM and 3.5 ± 0.06 μM, respectively; Figure 6D). Finally, fluid-phase FI cofactor assays, with purified reagents, indicated that C3b Asn1115 was more resistant to cleavage by human FI (with murine FH as cofactor) than WT murine C3b (Figure 6, E and F). These data are thus consistent with a mechanism whereby alternative pathway dysregulation in C3KI mice, arising from a perturbation of the binding site in C3b for the self/non-self-discriminatory C-terminal region of FH, is responsible for a TMA within the kidney and an aHUS-like phenotype in the mouse.

### Reversing complement dysregulation in C3KI mice.

The data presented above suggest that C3KI mice provide a unique model of complement-mediated disease that arises from a gain-of-function mutation rather than a loss-of-function change in complement regulators. The current gold standard treatment for complement-mediated aHUS in humans is eculizumab. We reasoned that treatment of C3KI mice with a C5-blocking mAb should protect them from developing aHUS.

**Figure 7:**
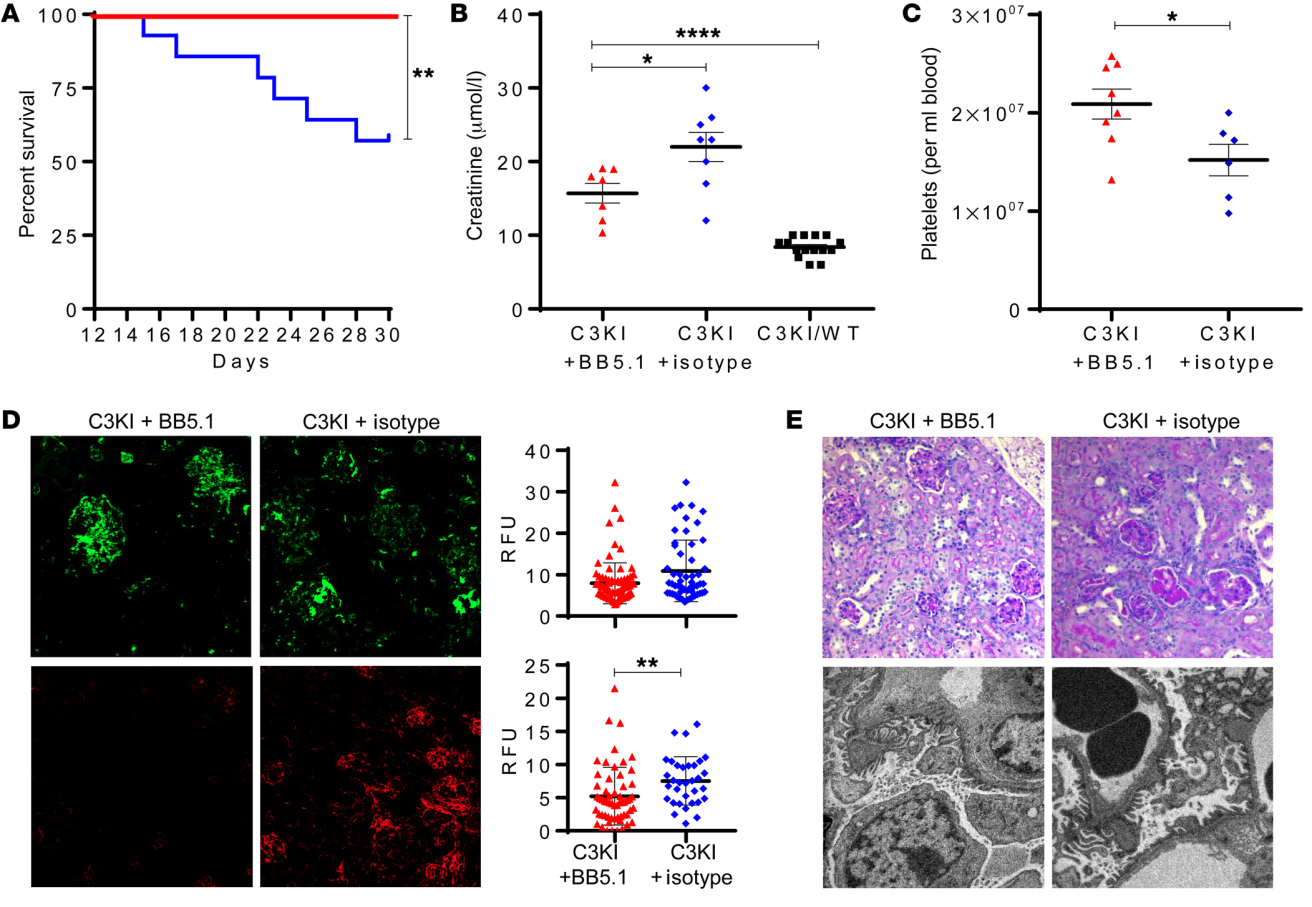
Treatment of the C3KI mice. (**A**) Kaplan-Meier survival curve analysis showing 100% survival in C3KI animals treated with BB5.1 (*n* = 8), in comparison to isotype control–treated animals (*n* = 11). (**B**) Where available, plasma creatinine values were analyzed. Shown are results for 7 C3KI mice treated with BB5.1 (C3KI + BB5.1, red triangles), 8 C3KI mice treated with isotype control antibody (C3KI + isotype, blue diamonds), and 15 untreated C3KI/WT mice (black squares). (**C**) Platelet counts of 8 C3KI mice treated with BB5.1 and 6 C3KI mice treated with isotype control antibody. Absolute numbers were provided by use of cell count beads and flow cytometry. (**D**) Frozen kidney sections were collected from C3KI mice treated with BB5.1 or isotype control antibody and stained for glomerular C3 (anti-C3, upper panels, original magnification, ×20) and C9 deposition (anti-C9, lower panels, original magnification, ×10). Images are representative of 5 images per animal analyzed. (**E**) Top panels: PAS-stained sections from C3KI mice treated with BB5.1 and IgG control. Histology demonstrates features of a TMA. Original magnification, ×20. Representative of 8 BB5.1-treated animals and 6 IgG control-treated animals. Bottom panels: Electron microscopy images of BB5.1-treated animals and IgG control showing foot process effacement and subtle areas of subendothelial lucency. Original magnification, ×5000 (BB5.1), ×6000 (IgG). Representative of 2 C3KI BB5.1-treated and 2 C3KI IgG control–treated mice. *P < 0.05, **P < 0.005, ****P < 0.0001 using unpaired *t* test with Welch’s correction.

Therefore, we injected P13–P30 C3KI mice with 50 μg/g BB5.1 (a C5-inhibiting mAb, with dosing based on previous studies; refs. 44, 45), for up to 2 weeks and compared them with C3KI mice dosed with isotype-control antibody (mouse IgG_1_/PBS). While 55% (6 of 11) of mice treated with the control antibody were removed from the study due to poor health, none of the mice treated with BB5.1 (0 of 8) succumbed to disease (Figure 7A). Failure to thrive, as indicated by low weight gain, was also partially reversed in BB5.1-treated mice compared with isotype controls (Supplemental Figure 9). Creatinine values were lower in the BB5.1 treatment arm, reflecting attenuation of renal injury, or else signs of renal recovery (Figure 7B). Platelet counts were significantly higher in the animals treated with BB5.1 (Figure 7C). C3 immunofluorescence appeared similar in both treatment arms, but C9 glomerular deposition was lower in the BB5.1 treatment arm (Figure 7D). Despite the marked clinical recovery in the BB5.1-treated cohort, histological analysis revealed only subtle improvement over the treatment period, with some persisting pathological features in both treated and control cohorts (Figure 7E). In general, an improvement in hematological outcomes for C3KI mice was achieved by C5 inhibition, consistent with a major role for the complement cascade in this mouse model of aHUS.

### Genetic deficiency in C5 protects mice from all kidney disease.

**Figure 8:**
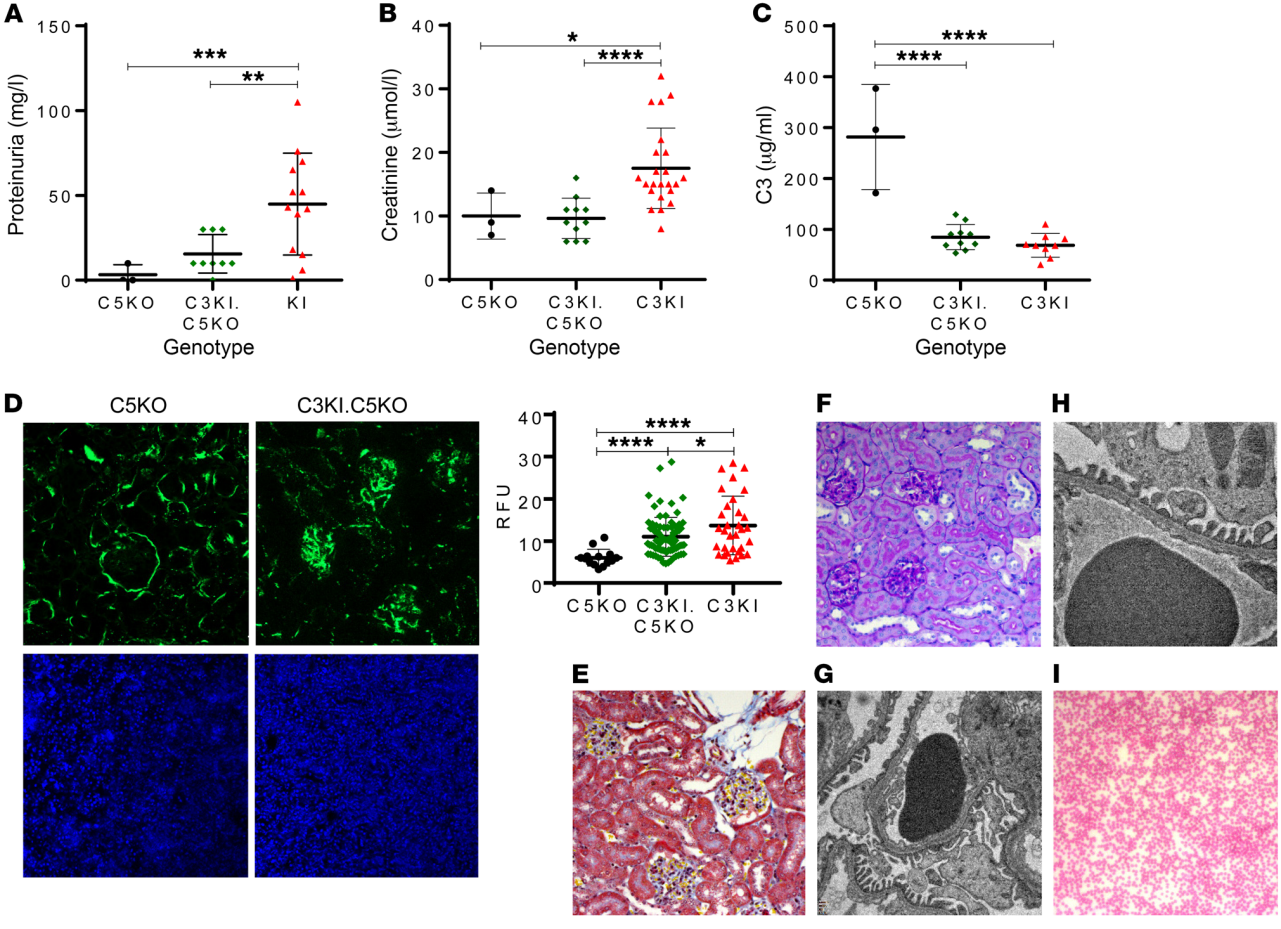
C3KI phenotype is rescued through genetic deletion of complement C5. (**A**) Proteinuria measurements of urine collected from 3 C5KO mice premortem (black circles) and, 9 C3KI.C5KO mice at 6 months of age (green diamonds) as well as, 12 C3KI mice on P21–P28 (red triangles). (**B**) Creatinine values obtained from 3 C5KO mice, 10 C3KI.C5KO mice at 6 months old, and 19 C3KI mice. (**C**) Plasma C3 levels. (**D**) Immunofluorescence of glomerular C3 deposition (upper panels; original magnification, ×20), with densitometry analysis of C3 deposition to the right, and with anti-C9 antibody (lower panels; original magnification, ×10). (**E**) MSB stain of C3KI.C5KO 6-month-old mice; original magnification, ×20 (representative of *n* = 8 examined). (**F**) PAS-stained sections of C3KI.C5KO 6-month-old mice showing normal glomeruli; original magnification, ×20 (representative of *n* = 8 examined). (**G**) Electron microscopy of a 6-month-old C3KI.C5KO mouse showing normal glomerular basement membrane and no evidence of dense deposits; original magnification, ×6000 (representative of *n* = 4 examined). (**H**) Electron microscopy of a 6-month-old WT mouse showing normal glomerular basement membranes; original magnification, ×10,000 (representative of *n* = 4 examined). (**I**) Diff-Quik–stained blood film of a C3KI.C5KO 6-month-old mouse showing no evidence of fragmented red blood cells; original magnification, ×10 (representative of *n* = 8 examined). *P < 0.05, **P < 0.01, ***P < 0.001, ****P < 0.0001. Unpaired *t* test (**C**) with Welch’s correction (**A**, **B**, and **D**).

To further validate the model and provide translational insight into long-term C5 blockade, we backcrossed the C3KI onto the C5-deficient mice (C5KO). As expected there was 100% survival of the C3KI.C5KO mice. Two cohorts of mice were tracked and culled at 2 and 6 months, respectively. No evidence of proteinuria was detected in urine collected before mice were culled in either cohort (Figure 8A and Supplemental Figure 10A), and creatinine levels were similar to those in age- and littermate-matched controls (Figure 8B and Supplemental Figure 10B). Collectively, these observations indicate a lack of chronic kidney disease. Plasma C3 levels remained low in C3KI.C5KO mice (118 ± 6 μg/ml) (Supplemental Figure 10C). This was particularly evident in the 6-month-old animals (84 ± 8 μg/ml) when compared with C3KI mice (68 ± 8 μg/ml) or C5KO mice (281 ± 60 μg/ml) (Figure 8C). Nevertheless, levels of C3 in the C3KI.C5KO mice were significantly (*P* < 0.0001) higher than the levels reported in the FH-KO mice (current model of C3 glomerulopathy), where all the fluid-phase C3 had been consumed (Figure 2C; ref. 41). As might be surmised from the low plasma C3 levels, glomerular C3 fragment deposition was still evident in both cohorts (Figure 8D and Supplemental Figure 10, D and E), while C9 staining was absent (Figure 8D, lower panels). The C3 fragment deposition in C3KI.C5KO mice did not appear to lead to morphological features of C3 glomerulopathy, as glomeruli appeared normal in the Martius scarlet blue– (MSB-) and PAS-stained sections (Figure 8, E and F, and Supplemental Figure 10, F–I). This was further validated using electron microscopy, whereby 6-month C3KI.C5KO mice showed healthy glomeruli and no evidence of electron-dense deposits (Figure 8G), similar to aged-matched WT (Figure 8H) and in contrast to 6-month FH-KO animals, which showed pathological features of C3 glomerulopathy (Supplemental Figure 10J). Peripheral blood smears showed no evidence of schistocytes in the C3KI.C5KO mice (Figure 8I and Supplemental Figure 10K).

## Discussion

This study demonstrates that transferring a disease-associated single-nucleotide substitution in *C3*, present in a family within the NRCTC aHUS cohort, to murine *C3* faithfully recapitulated the salient features of the human disease. Indeed, it could be argued that the resultant mouse model of aHUS, with respect to disease pathology, represents a more faithful animal model of aHUS-like disease than those generated to date (24, 36, 37), and this makes it particularly relevant for the testing of new anti-complement therapies. The data presented herein support the prevailing view that aHUS arises primarily due to deficient protection of host cellular and extracellular matrix surfaces from complement activation. This aHUS mouse model, based on a C3 gain-of-function amino acid substitution, offers opportunities to further dissect the precise role of complement and other factors that drive aHUS in humans. The relative health of the heterozygous mice (C3KI/WT) despite markedly increased complement turnover also offers the potential to investigate how C3 activity shapes the progression of other complex genetic disorders, such as rheumatic and neurological conditions, including AMD. In short, the development of this precision, rapid, and spontaneous model of complement dysregulation, in conjunction with subsequent strategic backcrosses, has the potential to provide platforms to carry out preclinical testing of drugs (anti-complement in particular) for the treatment of patients with a wide variety of complement-mediated diseases.

FHΔSCR16–20–transgenic mice (24) established that deletion of the FH C-terminus and its self-surface-binding capabilities, combined with uncompromised FH N-terminus–mediated control of C3 depletion in fluid phase, allows development of an aHUS-like disease. This study was fully consistent with the discovery that most (although not all) disease-associated mutations in human FH cluster within SCRs 19–20, which are known to contain binding sites for C3d, the TED of C3b, and surface polyanions and to be crucial for distinguishing between self- and non-self-surfaces (46). Such a truncation of FH has, however, not been identified in humans.

In contrast, the present model derives from a single mutation in a murine gene that maps directly to the equivalent disease-linked mutation in humans (26). Our starting point was the discovery that mutations in human *C3* associated with aHUS encode amino acid substitutions that, in C3b, occur throughout the binding regions for FH, although there is a bias toward the region that binds to FH SCRs 19–20 (double the number found in the rest of C3), i.e., the TED of C3b. This clustering of aHUS-linked mutations in C3 is not as prominent as it is in FH, in which there is a “hot spot” of aHUS-linked mutations within SCRs 19–20 (4 times the number found in the rest of FH) (21, 29, 47). There is accumulating evidence that mutations that affect the functional properties of C3/C3b are associated not just with aHUS but also with membranoproliferative glomerulonephritis (MPGN)/C3G (39, 48–50), and defining that link remains of keen interest.

While we initially surveyed in vitro the functions of multiple candidate mutations in recombinant human C3, we were drawn to D1115N, since it maps to the binding interface between the TED of C3b and SCRs 19–20 of FH (38) but also results in complete loss of MCP binding when MCP is coated on a solid surface such as a sensor chip or ELISA well (26, 29). Our own analysis with recombinant human proteins, in which WT or variant C3(H_2_0) was attached to a CM5 Biacore chip and FH or MCP used as analyte, confirmed these findings (Supplemental Figure 1). Reduced FH binding was also observed in both the human and mouse proteins (Figure 6 and Supplemental Figures 1, 4, and 5). The clear affinity reductions observed in SPR studies of both recombinant and ex vivo purified C3b Asn1115 are consistent with loss of an important C-terminal binding interaction but retention of the other FH (i.e., SCRs 1–5)-binding sites. Indeed, the affinity of full-length mouse FH binding to rmC3b Asn1115 was almost identical to that for recombinant mFH SCRs 1–5 alone (or mouse Crry), and the loss of binding for human FH SCRs 19–20 appears to confirm the mechanism (Figure 6), consistent with the studies by Morgan et al. (38). These data suggest that the D1115N substitution of murine C3b primarily perturbs interactions with the C-terminus of FH and hence the ability of C3b Asn1115 to be regulated by FH and FI. This is somewhat surprising, based on the deficit in MCP binding noted with this change (Supplemental Figure 1) (26, 29). However, it is clear that the mutation has only subtle effects, if any, on the interaction of mouse C3b with Crry or the N-terminus of FH. This likely reflects differences in the contact residues/structures of mouse C3 in the context of Crry compared with human C3 in the context of MCP, but it may give important information regarding the pathogenic burden of the loss of interaction with MCP versus FH. Clinically, MCP mutations rarely progress to end-stage renal disease (ESRD); this is in contrast to the poor renal prognosis associated with FH and C3 mutations (4, 15, 16). A failure of FH to engage fully with the TED is expected to be particularly detrimental to protection of host cell surfaces from excess complement activation. We also found evidence that C3b Asn1115 is marginally more resistant than WT C3b to fluid-phase FH- and FI-mediated cleavage (Figure 6E and Supplemental Figure 4). This, in concert with the MCP deficiency, apparently resulted in lower FB levels in one of the patients; in the mice, there was also evidence of increased FB consumption despite normal interaction with Crry (Figure 1 and Supplemental Figure 7C). Interestingly, we also detected an increase in FH levels in the plasma of C3KI mice. This suggests a compensatory upregulation of *CFH* expression in an attempt to regulate the mutant C3b or that *CFH* expression can be modulated by the C3-driven inflammatory response. This observation is consistent with the mouse FH W1206R model of TMA, in which elevated FH levels were also observed (37). Notably, both NRCTC patients with the C3 Asn1115 mutation possessed plasma levels of FH above the normal range on the majority of readings, reaffirming the parallels of our model to human disease.

It is clear from previous work with transgenic mice that a more substantial plasma C3 depletion than that seen in the C3KI mice may lead to the alternative phenotype of MPGN/C3G (36, 41, 47). Moreover, normal fluid-phase regulation is preserved in all of the mouse models of aHUS reported to date (24, 37, 51). The phenotype we obtained is that of aHUS, as evidenced by the histological features of a renal TMA rather than C3G. We therefore infer that any enhanced resistance to fluid-phase cleavage by FI of C3b Asn1115 is not a major contributor to the disease-like symptoms observed in our model. Lower plasma C3 levels are seen in many aHUS humans with C3 mutations (26, 29). The lower C3 levels in our murine model are fully consistent with previously published mouse models of a TMA (24, 37).

Thus, we can conclude that in the C3KI mouse, once C3b Asn1115 is deposited on a surface, a positive feedback process can operate that is neither controllable by FH nor restricted by a shortage of C3. This leads to extensive C3b deposition and activation of the terminal pathway, as evidenced from our observation of C5 consumption coupled with glomerular C9 deposition (Figure 5, B and C). Thus, our data emphasize the delicate balance of complement regulation; disease develops when the balance is disturbed and the threshold of regulatory ability is breached. Our C3KI animals retain the C3 “resources” to overwhelm complement regulation at the cell surface despite plasma C3 levels being lower than those in their WT counterparts.

Our model of C3 convertase dysregulation has parallels with that in a study by the Song group, in which the aHUS-linked single amino acid substitution W1206R was transferred from human FH into murine FH (37). Homozygous FH W1206R mice developed a renal phenotype consistent with aHUS, but a subtantial proportion of animals also demonstrated an aggressive TMA, with notable thrombus found in multiple organs. Analysis of key organs in C3KI animals revealed no evidence of large thrombi or microthrombi, although we did not perform an exhaustive search. Across our study, 3 homozygous mice (of ~45 analyzed) showed symptoms consistent with stroke, but this was always in the clinical context of severe kidney failure and likely driven by hypertension. Thus, in these two models of aHUS, the mechanisms driving the fatal outcome likely differ substantially.

Given the successful use of C5 inhibition in aHUS patients (3, 35, 52–54) coupled with previous work showing that the disease is dependent on C5 (25), we sought to further validate our model through use of the murine C5 inhibitor BB5.1. As predicted, this rescued the phenotype, with 100% survival in C3KI mice at 30 days postpartum. While C3 fragment deposition in the kidney of BB5.1-treated mice appeared unchanged, there was a reduction in glomerular C9 deposition. This suggests that C5a and/or MAC are critical to the endothelial dysfunction and subsequent development of a TMA. Unfortunately, our analysis of kidney morphology in the treated animals was not as unequivocal, as it showed that renal pathology remained. However, it is likely that more time is necessary to allow for full histological recovery. Therefore, we reasoned that extending our treatment study (mirroring clinical management) would lead to histological improvement, given the dramatic clinical improvement observed in the treatment cohort; thus, a pragmatic extension to this work was then to analyze the long-term effects of C5 blockade using the C5-knockout animals.

Eculizumab has revolutionized the treatment of patients with aHUS; however, we do not yet know nor will we know for some time what the long-term effects of this drug will be in our patient population. While inhibition at the level of C5 arrests disease, it does not target the effect of a persisting dysregulated alternative pathway and subsequent upregulation of C3. This is a vital question to answer, as it translates to all complement mutations associated with aHUS and could directly affect longevity of treatment. It has been hypothesized that over time patients on C5-inhibiting therapy may revert to a C3G due to sustained C3 activation. Our 6-month mouse cohort provides us with what we believe to be the clearest evidence to date that long-term C5 inhibition is unlikely to result in an alternative complement-mediated renal disease, despite the presence of deposited C3 within the glomeruli. Six months of age in a mouse is the equivalent of 20 human years (55); thus, our data highlight that C5 blockade in patients for this time frame will be unlikely to lead to an alternative disease process.

Our results suggest that the present C3 gain-of-function mouse model of aHUS will allow evaluation of various complement-inhibiting therapies, for instance, targeting the ability of C5aR antagonists to reverse disease (56). This would allow the dissection of the independent roles of C5a and MAC in the pathogenesis of disease with a clarity not achievable by other currently available models. Furthermore, the C3 gain-of-function model offers the potential to assess therapies targeting the alternative pathway directly, such as homodimeric minimal FH (57), CR2-FH (58), and anti-properdin (59), in what we believe to be the most realistic model of complement genetic alterations to date. One major aim of our studies is to help test therapeutics that can mitigate the increased risk of infections posed by long-term use of eculizumab (34, 60–62).

We have shown that the C3KI mouse mirrors the aHUS clinical phenotype. A similar complement profile, i.e., low C3 levels and elevated FH levels in plasma, was seen in both patients and mice. Highly similar histological and biochemical phenotypes further underline the extent to which this model recapitulates the clinical phenotype in humans. Interestingly, in mice only homozygotes developed spontaneous disease early in life, while patients are heterozygous for C3 Asn1115. This is consistent with the notion that the genetic change predisposes to the human disease, but an environmental trigger is needed for development of the disease (15, 20, 63). We tracked our C3KI/WT mice for 12 months, and to date there is no evidence that the animals developed disease, consistent with the environmental trigger hypothesis. While the absence of disease in the heterozygotes could be seen as a limitation of this study, we would argue that they represent a model to identify the factors beyond uncontrolled complement activation that drive aHUS, i.e., the C3KI/WT mice can be used to investigate a range of potential environmental and dietary triggers that can precipitate aHUS. Collectively, our spontaneous and likely inducible model will enable testing of a variety of complement-inhibiting therapies, and importantly, testing of withdrawal of therapy and remission monitoring. This has the potential to culminate in a wealth of translational data that will directly inform future clinical trials and targeted therapies, with the overall aim of outcome of improving outcome in patients with complement-mediated diseases.

In summary, our mouse model of aHUS, engineered by using a single point mutation in C3, recapitulates the clinical phenotype found in humans. This C3 mutation results in systemic complement activation and endothelial dysfunction, culminating in a renal TMA. Our model provides the opportunity to investigate the roles played by various players downstream of C3 activation and is a test bed for future complement therapeutics. Developing a therapy to restore complement homeostasis would, in addition, be transferable to several other endothelial damage–mediated diseases for which therapy remains an unmet clinical need.

## Methods

### Mice

B6.C3^Tm1(D1115N)Oz/NCL^ (C3KI) mice were generated using standard knockin technology by Ozgene (see Supplemental Figure 5) and backcrossed onto constitutive Cre recombinase–expressing mice (B6^OZCre^; Ozgene) or constitutive FLPe recombinase–transgene mice (B6^OzFlpe^; Ozgene). These F_1_ C3KI/CRE mice were shipped to Newcastle and intercrossed to provide homozygous C3KI mice. Genotyping of C3KI mice was performed by PCR on digested ear notch tissue using the following strategy devised by Ozgene. Primer pairs 5′-CCTTCTCTTTCTGGAATTTGCCTG-3′ and 5′-CTTTGGTGACCCTGTCTGTTCC-3′ were used in 30 cycles of PCR (58°C annealing temperature), generating a 574-bp product in the case of native C3 and a 633-bp product for the knocked-in sequence. The 633-bp product was digested into fragments of 402 bp and 231 bp by incubating with restriction enzyme SpeI. Using a 2% agarose gel, amplicons were separated sufficiently to allow identification of WT or the C3KI gene carriers. The presence of the Cre recombinase gene was detected through use of the following PCR primers: 5′-ATTTACGGCGCTAAGGATGACTC-3′ and 5′-TTACACCTGTTCAATTCCCCTG-3′, which generated a 680-bp amplicon in 30 cycles of PCR (58°C annealing temperature) (designed at Ozgene). FH-knockout mice (FH-KO, ref. 41) were generated by our research team. C3KI mice were backcrossed onto the previously described C5-deficient mice (C5KO; via Matthew Pickering) (25). In brief, C3KI/WT were crossed with C5KO mice. Pups were then all C5KO/WT, and those that were also C3KI/WT were crossed with C5KO mice. Pups from this cross were screened by PCR using DBA primers: 5′-CACGATAATGGGAGTCATCTGCG-3′ and 5′-AAGTTGGAGTGTGGTCTTTGGGCC-3′, followed by a HindIII digest to distinguish between WT, +/–, and –/– genotypes. Pups that were C3KI/WT.C5KO were then crossed to produce C3KI.C5KO mice.

### Protein purification

#### Murine C3 from plasma.

Mouse C3 for biochemical analysis was purified from mouse plasma according to the method of Van den Berg et al. (64). Briefly, 10 ml mouse serum was precipitated using polyethylene glycol (PEG) followed by application onto a Mono Q anion-exchange column (GE, HR5/5) and gel filtration chromatography using a HiLoad 16/600 Superdex 200 pg column. WT and mutant mouse C3 protein (C3 Asn1115) concentrations were calculated by measuring absorbance at 280 nm using an extinction coefficient of 183,033 and a molecular weight of 186.48 kDa as determined from the mature WT amino acid sequence (see Supplemental Figure 2A) using the ExPASy ProtParam tool. Reducing and nonreducing SDS-PAGE gels were used to provide evidence of homogeneity.

#### Mouse FH.

Murine FH was purified from 20 ml mouse serum using a previously generated mAb (2A5, anti-mouse FH, a gift from Claire Harris, Newcastle University) affinity chromatography resin using an ÄKTA Start (GE), followed by gel filtration chromatography on HiLoad 16/600 Superdex 200 pg column.

### SDS-PAGE and Western blotting

Gels with the appropriate percentage of acrylamide were freshly prepared for each SDS-PAGE run. Samples containing recombinant or plasma-derived C3 were run under both nonreducing and reducing conditions. Protein bands were either stained using Coomassie blue or were transferred to nitrocellulose. The nitrocellulose was blocked in 5% w/v nonfat milk in PBS overnight at 4°C, then exposed to a polyclonal goat anti–mouse C3 antibody–HRP (1:10,000; catalog 55557, lot 06803, MP Biomedicals). After extensive washing in 0.01% v/v Tween in PBS, the nitrocellulose was then developed using Bio-Rad Clarity Western ECL substrate, followed by detection/analysis on the Odyssey FC imaging system (LI-COR).

### Fluid-phase cofactor activity assays

See Supplemental Methods.

### SPR

All experiments were conducted on a BIAcore S200. Each version of murine C3 was converted to C3b, via incubation with purified human FB and FD (at molar concentrations 50 and 1000 times less, respectively, than that of C3) at 37°C in PBS supplemented with 1 mM MgCl_2_ for 1 hour. The incubated samples were gel filtrated using a Superdex 200 10/300 GL column; C3b-containing fractions were pooled and concentrated. Each version of murine C3b (i.e., WT C3b and C3b Asn1115) was immobilized (930 ± 10 RU) on separate flow cells of a CM5 chip using standard amine coupling. A 2-fold dilution series of purified murine FH (10 to 0 μM), recombinant murine FH SCRs 1–5 (14.6 to 0 μM), recombinant human FH SCRs 19–20 (20 to 0 μM), or recombinant murine Crry SCRs 1–5 (10 to 0 μM) was made in HBST buffer (10 mM HEPES, 150 mM NaCl, and 0.005% Tween-20, pH 7.4) and flowed across either chip surface in the same buffer. All analytes were injected in duplicate (30 μl/minutes for 200 seconds), followed by running buffer for 300 seconds and a regeneration phase involving injection of regeneration buffer (10 mM sodium acetate, 1 M NaCl, pH 4.5) for 60 seconds. The equilibrium dissociation constant, *K*_D_, and standard error were calculated using the steady-state model in BIAcore S200 Evaluation Software.

### ELISA

#### Murine C3.

To determine mouse C3 levels, mAb 11H9 (recognizing both intact C3 and its cleaved products C3b, iC3b, C3d, and C3dg; catalog HM1045, Hycult Biotech via Cambridge Bioscience) was coated on each well of a Nunc MaxiSorp flat-bottom ELISA plate (20 ng/well) to capture mouse C3. Then 50 μl of a 1:800-diluted sample was applied for analysis. Bound C3 was detected through use of an HRP-conjugated goat polyclonal anti–mouse C3 antibody (1:25,000, catalog 55557, lot 06803, MP Biomedicals). This dilution biases the assay to detect bound intact C3. The ELISA was developed with TMB. The C3 concentrations were interpreted based on a standard curve generated using purified WT mouse C3 and interpolated using GraphPad Prism software.

#### FH.

To measure FH present in mouse plasma, ELISA plates (Nunc) were coated with monoclonal anti-FH (2A5) at 1 μg/ml. After overnight incubation at 4°C, plates were blocked with 1% w/v BSA at room temperature for 1 hour. After washing, plates were incubated with diluted mouse plasma (1:1000) for 1 hour at room temperature. Plates were washed and then incubated with sheep anti–mouse FH (1:5000, Abcam Ab8842) for 1 hour at room temperature and then detected by adding donkey anti-sheep HRP-conjugated antibody (1:5000, 713-035-147, lot 125113, Jackson ImmunoResearch Laboratories Inc. via Stratech Scientific) for 1 hour at room temperature. A standard curve was compiled using the known concentration of purified mouse CFH.

#### C5.

To measure C5 present in mouse plasma, ELISA plates were coated with 1 μg/ml BB5.1 (anti-C5, catalog HM1073, Hycult Biotech via Cambridge Biosciences). After overnight incubation at 4°C, plates were blocked with 1% w/v BSA at room temperature for 1 hour and then washed. Plates were then incubated with diluted mouse plasma (1:1000) for 1 hour, washed, and then incubated with goat anti–human C5 (1:1000, Quidel A306, 073139). Plates were then incubated with donkey anti-goat HRP-conjugated antibody (1:5000, 705-036-147, lot 124700 Jackson ImmunoResearch Laboratories Inc. via Stratech Scientific). After a final wash, the plate was developed with TMB. Samples were standardized to WT control.

### Assessment of murine renal function and hematological parameters

Plasma creatinine was measured by collecting plasma by cardiac puncture into EDTA and subsequently measured on a Cobas 602 analyzer in the Newcastle Hospitals NHS laboratories. Plasma urea was measured using a urea assay kit (MAK006, Sigma-Aldrich). Mouse urine was collected and measured for hematuria and proteinuria using Hema-Combistix (Siemens/Bayer). Platelet analysis was performed by flow cytometry. Briefly, 10 μl EDTA-blood was mixed with 400 μl red cell lysis buffer (Sigma-Aldrich) and incubated for 10 minutes at room temperature. An aliquot of 5 μl was then transferred to 1 ml flow buffer (PBS containing 5% w/v BSA, 1 mM EDTA, 0.1% w/v Na azide) and washed at 1000 *g* for 5 minutes, 3 times, and resuspended in 200 μl flow buffer containing 1:400 CD41-FITC, 1:800 CD61-PE, and 1:800 CD62P- BV421 (catalog 553848, 553347, and 564289, respectively, BD Biosciences). Samples were incubated for an hour on ice in the dark before washing 3 times with 3 ml flow buffer (1000 *g* for 5 minutes). Finally, samples were resuspended in 250 μl of 1% w/v paraformaldehyde/flow buffer with 25 μl CountBright beads (i.e., 2.5 × 10^4^ beads per tube; Invitrogen). Data were collected using a FACSCanto flow cytometer and analyzed on FCS Express, version 6 (De Novo Software). Alternatively, platelets were analyzed by using an improved Neubauer hemocytometer through addition of 25 μl of the red cell lysed solution (as above) and allowing the sample to settle for 10 minutes before counting as per the manufacturer’s instructions.

### Histological analysis

#### Light microscopy.

Kidneys were harvested and fixed in 10% buffered formalin solution. Following fixation, the tissues were embedded in paraffin, then cut in 3-μm sections. Sections were stained with PAS reagent, then examined by light microscopy. Newcastle Hospitals NHS pathology laboratories stained for fibrin using a standard MSB stain protocol. Sections were blinded to avoid interpreter bias.

#### Immunofluorescence.

Mouse kidneys were snap-frozen in isopentane (pre-cooled in liquid nitrogen) and stored at –80°C in individual containers. Five-micrometer cyrosections from mouse kidneys were mounted on a Shandon ColorFrost Plus microscope slide (Thermo Fisher Scientific), before fixing in acetone and stored in –80°C. The thawed tissue sections were blocked for an hour with 60 μl of 20% (v/v) goat serum in PBS, then detected with 60 μl FITC-conjugated goat polyclonal anti–mouse C3 antibody (catalog 0855500, MP Biomedicals, 1:100). After repeated washing with PBS, the tissue sections were stained with DAPI (catalog H-1200, Vector Laboratories) and covered with glass coverslips. Fluorescence images were taken at ×10 or ×20 magnification utilizing a Zeiss Axio Imager II.

For C9 immunofluorescence, sections were blocked with rabbit and goat serum, followed by rat anti–mouse CD16/CD32 Fc block (catalog 553141, BD Pharmingen 1:100) in 2% w/v BSA in PBS for 1 hour at room temperature. After washing, sections were incubated with rabbit anti–rat C9 (1:75, a gift from B.P. Morgan, Cardiff University, Cardiff, United Kingdom) overnight at 4°C. The next day, sections were washed, then incubated with goat anti-rabbit Alexa Fluor 594 (1:100, catalog A-11012, Abcam). Finally, the sections were washed, mounted in DAPI, and imaged as above. Immunofluorescence images were analyzed in ImageJ (NIH) and expressed as relative fluorescence units (RFU).

#### Electron microscopy.

Small pieces of cortical tissue (1 mm^3^) were fixed in 2% w/v glutaraldehyde in 0.1 M cacodylate buffer, post-fixed in osmium tetroxide, dehydrated in a graded series of acetone, and embedded in Taab epoxy resin. Ultrathin sections (70 nm) were picked up on copper grids, stained with uranyl acetate and lead citrate, and examined using a Philips CM100 transmission electron microscope (EM Research Services, Newcastle University).

#### Availability of data and materials.

The C3KI conditional/constitutive mouse and recombinant mouse C3 plasmid/protein are available through a material transfer agreement (MTA) with Newcastle University.

### Statistics

Data were analyzed using GraphPad Prism 7.00 for Windows (GraphPad Software). Densitometry was carried out using Image studio version 5.2 (LI-COR). Groups were compared at each time point using 1- or 2-way ANOVA with Bonferroni’s multiple-comparisons test as indicated. A *P* value less than 0.05 was considered significant; *P < 0.05, **P < 0.01, ***P < 0.0005. Student’s *t* test was 2-tailed, and data are shown as mean ± SEM.

### Study approval

All animal experiments were approved by the ethics committee of the Comparative Biology Centre of Newcastle University and performed under the United Kingdom’s Home Office granted license PD86B3678. The clinical study involving subjects from the NRCTC was approved by the Northern and Yorkshire Multi-Centre Research Ethics Committee, and informed consent obtained in accordance with the Declaration of Helsinki.

## Author contributions

KJM and KSJ conceived, carried out, wrote, reviewed data for, and funded the study. YY, IYP, HD, KC, and HTC collected data, analyzed data, and edited the manuscript. MCP provided supervision and critical analysis, and edited the manuscript. DK and PNB carried out in silico analysis of mutant C3 molecules, provided reagents and supervision, and helped to write the manuscript. JPA and MKL provided reagents, supervision, and critical analysis, and edited the manuscript.

## Supplementary Material

Supplemental data

## Version 1 02/04/2019

Electronic publication

## Version 2 03/01/2019

Print issue publication
