# Supplementary material for: Hyperfunctional complement C3 promotes C5-dependent atypical hemolytic uremic syndrome in mice
Source: J Clin Invest. 2019 Feb 4;129(3):1061–75. doi: 10.1172/JCI99296 (PMC6391106; doi:10.1172/JCI99296)

Supplementary Materials.

Recombinant murine protein production.

Plasmid pDR2ΔEF1α was obtained from Dr I. Anegon (University of Nantes, Nantes, France) (65) and a new multiple-cloning site was engineered through restriction enzyme digestion of the plasmid with *Xba* I-HF and *Eco*R V (NEB, UK) and insertion of DNA oligonucleotides introducing *Nhe* I and *Not* I sites (see supplementary table 2). Oligonucleotides were heated to 95 ^{0}C in PCR buffer and allowed to cool to room temperature before being ligated into a pre-digested pDR2 plasmid, using T4 DNA ligase according to manufacturer's instructions (NEB, UK), and thus creating pDR2-nMCS-3. For recombinant mouse C3 (rmC3) production, a synthetic DNA construct was ordered (Eurofin MWG Operon, Germany). It contained a *Nhe* I site, bp -14 to bp 72 of m*C3* followed by the FLAG Tag sequence (see supplemental table 3), pp 104 to bp 390 of murine *C3* (m*C3*) (ending with the internal *Kpn* I site), a short six-bp linker (GGAGGA) then (beginning with the internal *Sal* I site) bp 4625 to 4988 m*C3* followed by a *PME* I site, a thrombin cleavage site, a 7x histidine tag (see supplementary table 3) and finally, the m*C3* stop codon, bp 4989 to 5032, plus a *Not* I restriction site. This synthetic construct was released from the supplied vector using the flanking *Nhe* I-HF and *No*t I-HF restriction sites and cloned into pDR2-nMCS-3. A *Kpn* I to *Sal* I fragment of mouse *C3* cDNA was then isolated from a commercially available Image clone (5134713), supplied by Source Bioscience (UK; IRAV p968G0388D; pCMV-Sport6) that contained the full mouse cDNA coding for *C3* (-42 to 5133 NCBI sequence reference BC043338.1 v NM_009778.3). Site-directed mutagenesis was carried out using the primers in supplementary table 1 and according to manufacturer's instructions (Quikchange XL, Stratagene, UK). Recombinant mouse FH SCRs 1-5 and minimal mouse Factor H (mini-mFH), SCR1-5 fused to SCR18-20, were PCR amplified from a pBluescript plasmid containing the DNA coding for full-length murine

*CFH (*a gift from Dr Elena Goicoechea de Jorge, Madrid, Spain) with flanking restriction sites, i.e. *Xba* I to *Bam* HI (plus 6x Histidine tag and stop, for mFH1-5) and *Bam* HI to 6x Histidine tag, stop, *Nhe* I (for minimal mouse FH), respectively. *Bam* HI-digested PCR products were ligated and used as template for the 5' *Xba* I and 3' *Nhe* I-containing primers. The resulting PCR product was double digested and ligated into cut and de-phosphorylated pDR2-nMCS-3. All plasmids were Sanger sequenced (using overlapping primers as illustrated for rmC3 in supplemental figure 2) before being transfected into Chinese hamster ovary (CHO) cells using the JetPEI reagent according to the manufacturer's (Polyplus transfection) instructions. Inclusion of hygromycin B (600 µg/ml) and limiting dilution allowed the selection of clonal transfectants. The various recombinant proteins were purified from tissue-culture supernatant using immobilized metal-ion or FLAG-tag affinity chromatography, as required, using a standard imidazole gradient (up to 500 mM) or 0.1 M glycine, pH 2.5, elution. The resultant recombinant mature mouse C3 containing the Flag/His-tags (rmC3) had a predicted molecular weight of 187247 Daltons, a predicted pI of 6.31, and a calculated extinction coefficient of 184525 as determined from the amino acid sequence using ExPASy ProtParam tool (https://web.expasy.org/protparam/). Purity was confirmed using 10% non-reducing and reducing SDS-PAGE (Supplementary data figure 3). The human FH19-20 fragment was produced in *Pichia Pastoris* as previously described (34). Recombinant soluble mouse CRRY (mCR1l[NM_013499.2]/Thrombin/10xHis) was designed in house and a plasmid generated by VectorBuilder (Cyagen Biosciences, CA, USA) before being transfected into CHO cells and then purified from tissue culture supernatant using HIS-tag affinity chromatography, using a standard imidazole gradient (up to 500 mM).

Expression of recombinant human C3 (rhC3,WT) or rhC3 Asn1115.

WT and p.D1115N human C3 plasmids were prepared previously (29). Plasmids were transformed and amplified into E. Coli DH5α, purified using a Maxiprep kit (QIAGEN). The mutant and WT C3 DNAs were transiently transfected into HEK 293F cells (Invitrogen) using polyethylenimine transfection reagent based on an established protocol (66), using FreeStyle 293 expression medium. 3 days after transfection, the supernatants were collected and adjusted to pH 7.5 using Trizma base solution (pH 9.0). The purification processes were based on (67), the pH adjusted supernatants were loaded on a 5 ml Hitrap Q column, the C3 containing fractions were identified and purified further via a mono-S cation exchange column. Concentrated C3 fractions were hydrolysed by incubating with 50 mM methylamine for 2 hours, at 37 degree, in PBS pH 8.2. The resulted C3(H_2O) was dialysed against HBST buffer prior the Biacore analysis. Human FH was purified from plasma via affinity chromatography using a method previously described. Purified MCP domain 1-4 was a prepared previously (29).

Each version of human hydrolysed C3 was immobilised (1000 ±50 RU) on separate flow cells of a CM5 chip using standard amine coupling. A two-fold dilution series of purified human FH (10 to 0 μM) or recombinant human MCP SCR 1-4 (2.984 to 0 μM) were made in HBST buffer (10 mM HEPES, 150 mM NaCl, and 0.005% Tween 20, pH 7.4) and flowed across either chip surface in the same buffer. All analytes were injected in duplicate (30 μl/min for 190 *s*), followed by running buffer for 300 *s* and a regeneration phase involving injection of regeneration buffer (10 mM sodium acetate, 1 M NaCl, pH 4.5) for 60 *s*. The equilibrium dissociation constant K_D and standard error were calculated using steady-state model in BIAcore S200 Evaluation Software.

Fluid phase co-factor activity assays

For studies on recombinant mouse C3 (rmC3), 5 µg of affinity-purified rmC3b and mutants Val1072, Asn1115 were incubated with mouse mini-FH and serum-derived mouse complement factor I for increasing time at 37 ^{o}C. Reduced samples (1/10 diluted) were applied to 10% PAGE gels, Western blotted and exposed to an anti-His tag antibody (Penta-His 1/1000, cat. 34660; Qiagen) followed by 1/500 SA-HRPO. For experiments where C3 was isolated from mice, a 30-µl reaction volume was used. Either purified WT or murine C3 Asn1115 was converted to C3b as follows. In brief, 3 µg of either recombinant or plasma-derived murine C3 was incubated with 1 µg of human complement factor B, and 0.01 µg of human complement factor D in the presence of 5 mM $MgCl_2$ at 37 °C for one hour (human complement factor B and D purchased from Comptech, Texas, USA). After the reaction was stopped by the addition of 10 mM EDTA, plasma purified murine FH and human FI were added to final concentrations of 200 nM and 20 nM respectively. The reaction was incubated at 37 °C, and 4-µl sample was taken at 0, 5, 10, 20, 30, 40 and 60 min and analyzed by anti-murine C3 Western blotting.

References for supplementary methods (ordered per main article).

29. Schramm EC, Roumenina LT, Rybkine T, Chauvet S, Vieira-Martins P, Hue C, Maga T, Valoti E, Wilson V, Jokiranta S, et al. Mapping interactions between complement C3 and regulators using mutations in atypical hemolytic uremic syndrome. Blood. 2015;125(15):2359-69.

65. Charreau B, Tesson L, Menoret S, Buscail J, Soulillou JP, and Anegon I. Production of transgenic rats for human regulators of complement activation. Transplant Proc. 1997;29(3):1770.

66. Portolano N, Watson PJ, Fairall L, Millard CJ, Milano CP, Song Y, Cowley SM, and Schwabe JW. Recombinant protein expression for structural biology in HEK 293F suspension cells: a novel and accessible approach. J Vis Exp. 201492):e51897.

67. Alsenz J, Avila D, Huemer HP, Esparza I, Becherer JD, Kinoshita T, Wang Y, Oppermann S, and Lambris JD. Phylogeny of the third component of complement, C3: analysis of the conservation of human CR1, CR2, H, and B binding sites, concanavalin A binding sites, and thiolester bond in the C3 from different species. Dev Comp Immunol. 1992;16(1):63-

Supplementary Figure 1.

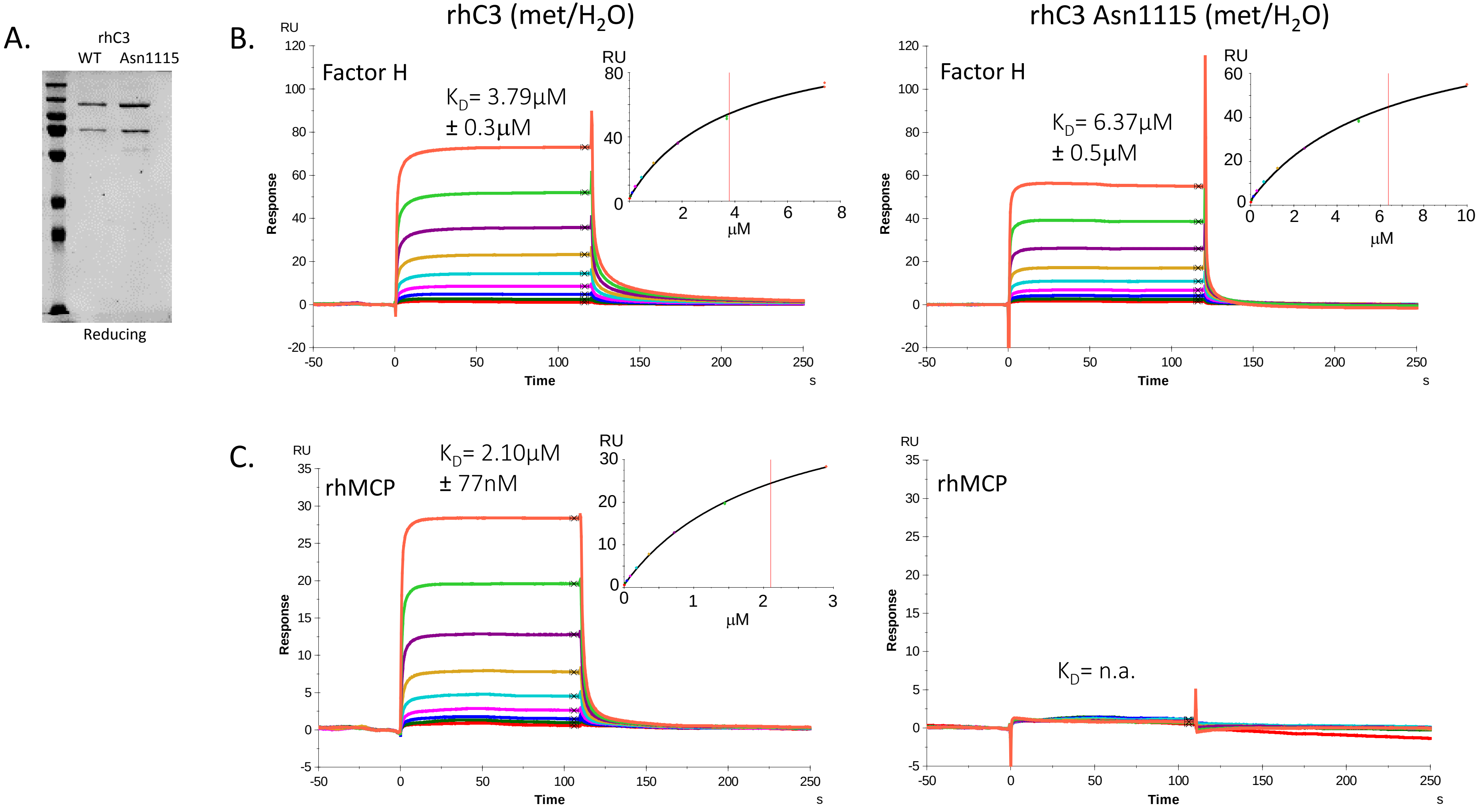

(A) A 10% reducing SDS-PAGE gel of purified recombinant human C3 (rhC3,WT) or rhC3Asn1115 produced via transient expression in HEK293 (Invitrogen, UK) is shown. SPR analysis was performed after methylamine treatment of the rhC3 variants and coupling to a CM5 biosensor chip (1000 ± 10 RU). Doubly diluted concentration series of (B) purified human FH (0 to 10 μM) or (C) rhMCP (0 to 3 μM), were flowed across either chip surface. The equilibrium dissociation constant K_D was calculated using steady state model in Biacore^{TM} evaluation package, indicated as the black vertical line.

Supplementary Figure 2A.

```
                24-aa signal sequence
Mouse   1     MGPASGSQLLVLLLLLASSPLALGIPMYSIITPNVLRLESEETIVLEAHDAQGDIPVTVT   60
Human   1     ...T..PS..--....THL.....S.........I........M..........V.....   58
                22-aa signal sequence

Mouse   61    VQDFL-KRQVLTSEKTVLTGASGHLRSVSIKIPASKEFNSDKEGHKYVTVVANFGETVVE   119 G79
Human   59    .H..PG.KL..S.......P.TN.MGN.TFT...NR..K.E.GRN.F...Q.T..TQ...   118 R80

Mouse   120   KAVMVSFQSGYLFIQTDKTIYTPGSTVLYRIFTVDNNLLPVGKTVVILIETPDGIPVKRD   179 T139
Human   119   .V.L..L...........................NHK.....R..MVN..N.E.....Q.   178 T140
....
Mouse   360   KTPKFFKPAMPFDLMVFVTNPDGSPASKVLVVTQGSNA-KALTQDDGVAKLSINTPNSRQ   418 P346
Human   359   ....Y...G..............YR.P.AV..EDTVQS...G..........HP.QK   418 P347
....
Mouse   539   GASGQREVVADSVWVDVKDSCIGTLVVK-GDPRDNHLAPGQQTTLRIEGNQGARVGLVAV   597 R567
Human   539   ......................V.S....S.QSE.RQPV...M..K...DH....V....   598 R570
....
                      cleavage
Mouse   658   DLECTKPAARRRRSVQLMERRMDKAGQYTDKGLRKCCEDGMRDIPMRYSCQRRARLITQG   715
Human   659   E.Q.PQ...........T.K....V.K.P-.E..........EN...F.....T.F.SL.   717
                      cleavage          ANA domain (C3a)
Mouse   718   ENCIKAFIDCCNHITKLREQHRRDHVLGLARSELEEDIIPEEDIISRSHFPQSWLWTIEE   777 R711
Human   718   .A.K.V.L....Y..E..R..A.ASH......N.D....A..N.V...E..E....NV.D   777 R713
....
Mouse   838   SVVRNEQVEIRAVLFNYREQEELKVRVELLHNPAFCSMATAKNRYFQTIKIPPKSSVAVP   897 Y830
Human   838   ..............Y...QNQ................L..T.R.HQ..VT......LS..   897 Y832

Mouse   898   YVIVPLKIGQQEVEVKAAVFNHFISDGVKKTLKVVPEGMRINKTVAIHTLDPEKLGQGGV   957 ΔDG
Human   898   .......T.L.........YH.......R.S.......I.M....VR......R..RE..   957 ΔDG
....
Mouse   1078  LTAYVVKVFSLAANLIAIDSHVLCGAVKWLILEKQKPDGVFQEDGPVIHQEMIGGFRNAK   1137 A1070, D1091
Human   1078  ............V.......Q.......................A..........L..NN   1137 A1072, D1093

Mouse   1138  EADVSLTAFVLIALQEARDICEGQVNSLPGSINKAGEYIEASYMNLQRPYTVAIAGYALA   1197 Q1137
Human   1138  .K.MA.......S....K....E.........T...DFL..N......S...........   1197 Q1139
....
Mouse   1438  FSNKNTLIIYLEKISHTEEDCLTFKVHQYFNVGLIQPGSVKVYSYYNLEESCTRFYHPEK   1497 H1440
Human   1438  ..DR.......D.V..S.D...A.........E.....A....A................   1497 H1442
....
Mouse   1618  TSYIIGKDTWVEHWPEAEECQDQKYQKQCEELGAFTESMVVYGCPN   1663
Human   1618  L...............ED....EEN....QD..........F....   1663
```

Pairwise alignment (BLAST) of selected sequences from mouse and human C3. Residue numbering includes signal sequences (labeled and shaded) but mutations (highlighted in magenta) are numbered (on the right), based on the mature mouse and human protein sequences as used in Figure 1B. The cleavage site (generating chains B and A of mature C3) and the ANA domain (excised during production from C3 of C3b) are labeled and shaded in red and green, respectively.

Supplementary Figure 2B. Alignment of C3 proteins and disease associated mutants

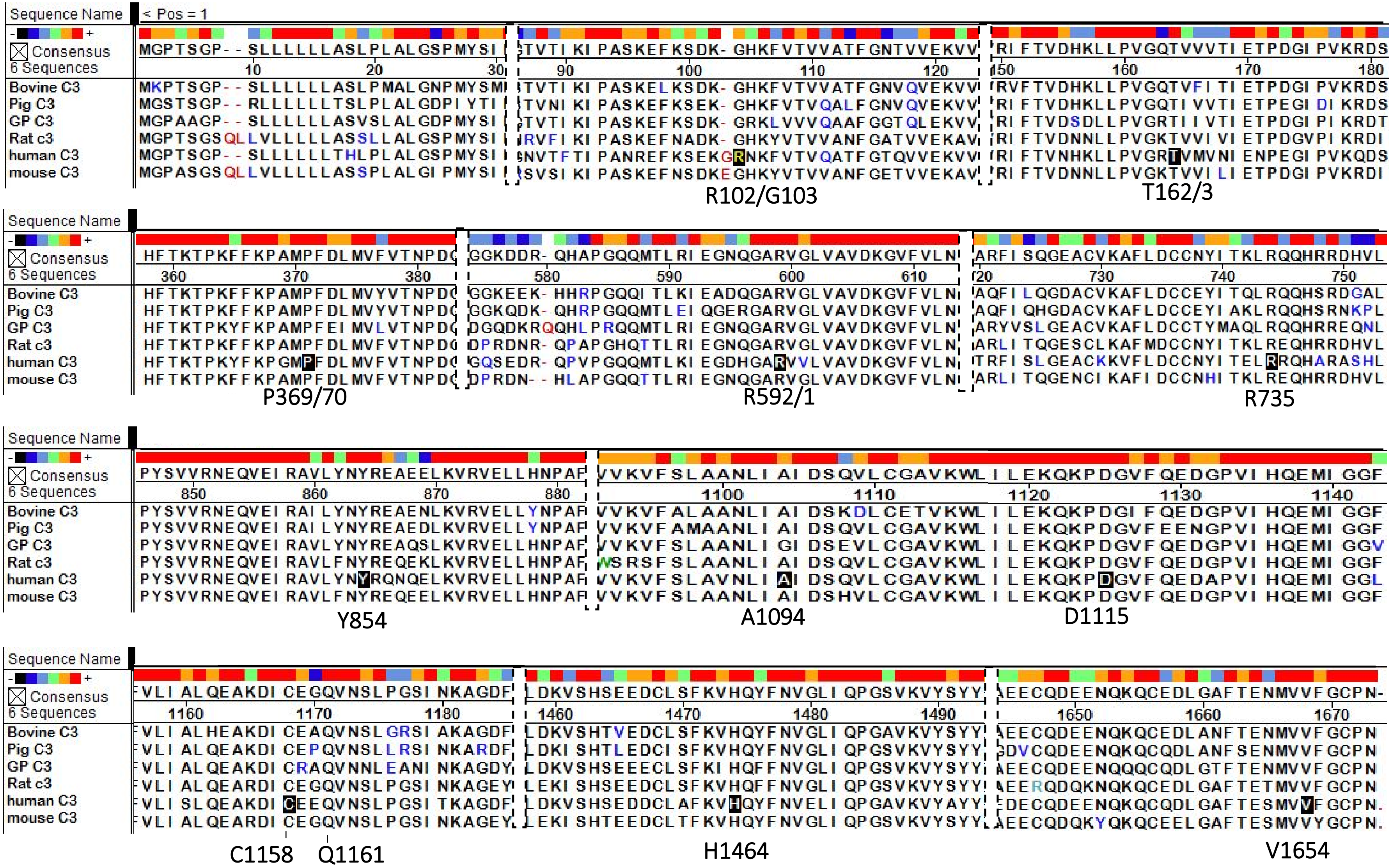

Pairwise alignment (BLAST) of selected sequences from various species. Bovine (Q2UVX4), Pig (P01025), Guinea Pig (GP;P12387), Rat (P01026), Human (P01024) and Mouse C3 (P01027) protein sequences taken from Uniprot (http://www.uniprot.org/uniprot/) were aligned using ClustralW in DNAstar. Met +1. Human position/mouse position of selected mutants are noted under aligned sequence.

Supplementary Figure 2C.

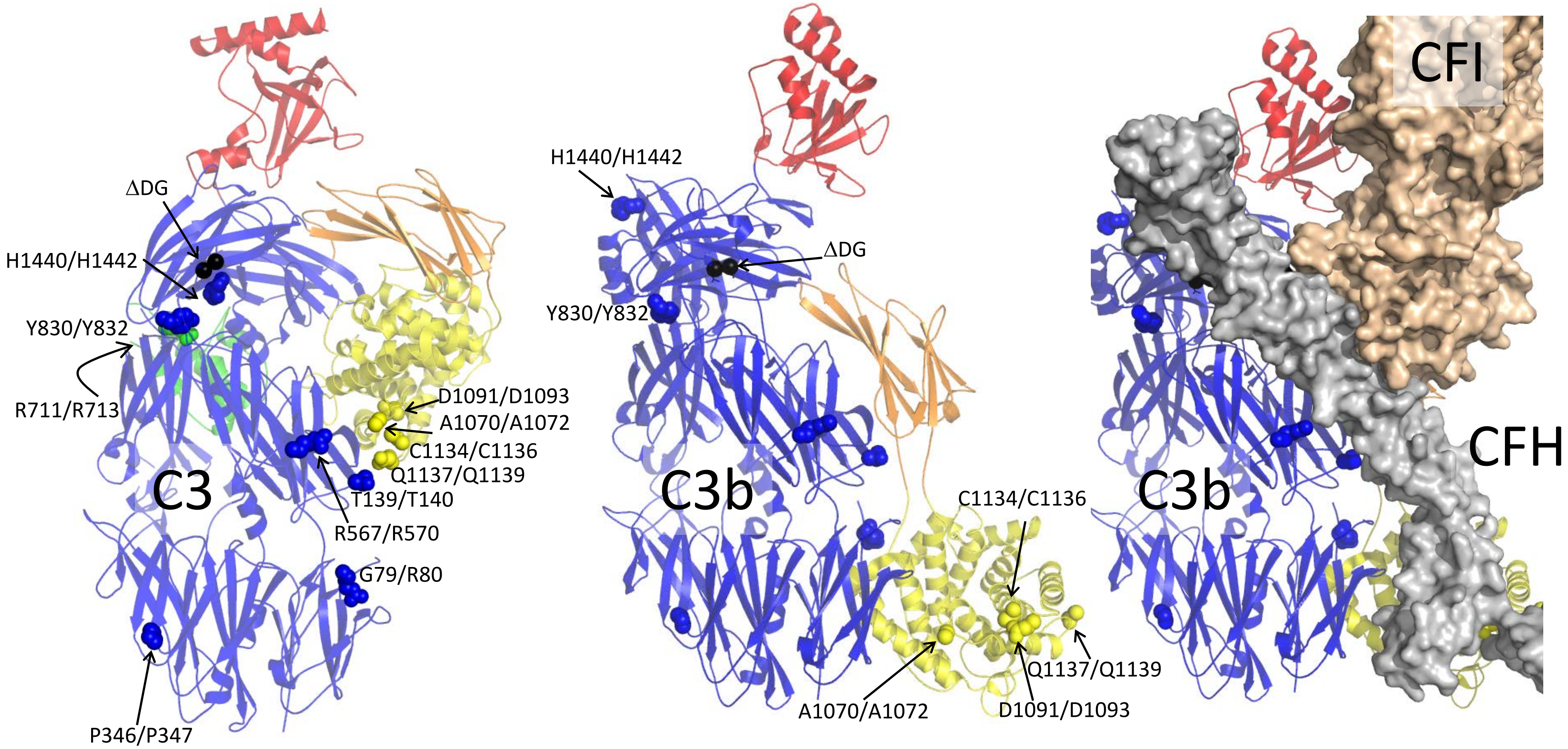

Locations of mutations in C3 and C3b. Representations of human C3 (pdb = 2a73), and of human C3b taken from the crystal structure of C3b in complex with "mini"-FH (FH SCR domains 1-4 and 19-20 joined by a flexible linker) and FI (pdb = 5o32). C3 and C3b are drawn as cartoons and mini-FH and FI are represented by their surfaces. FI and mini-FH are omitted from the central panel, for clarity. The side-chains of mutated residues are shown as spheres and numbered as in the mature sequences of mouse/human C3; except for the Asp-Gly deletion identified by black spheres representing Ca atoms. The C345C domains are red, the CUB domains are orange, the ANA domain (in C3 only) is green and the thioester domains are yellow; other C3 and C3b domains are blue.

Supplementary Figure 3.

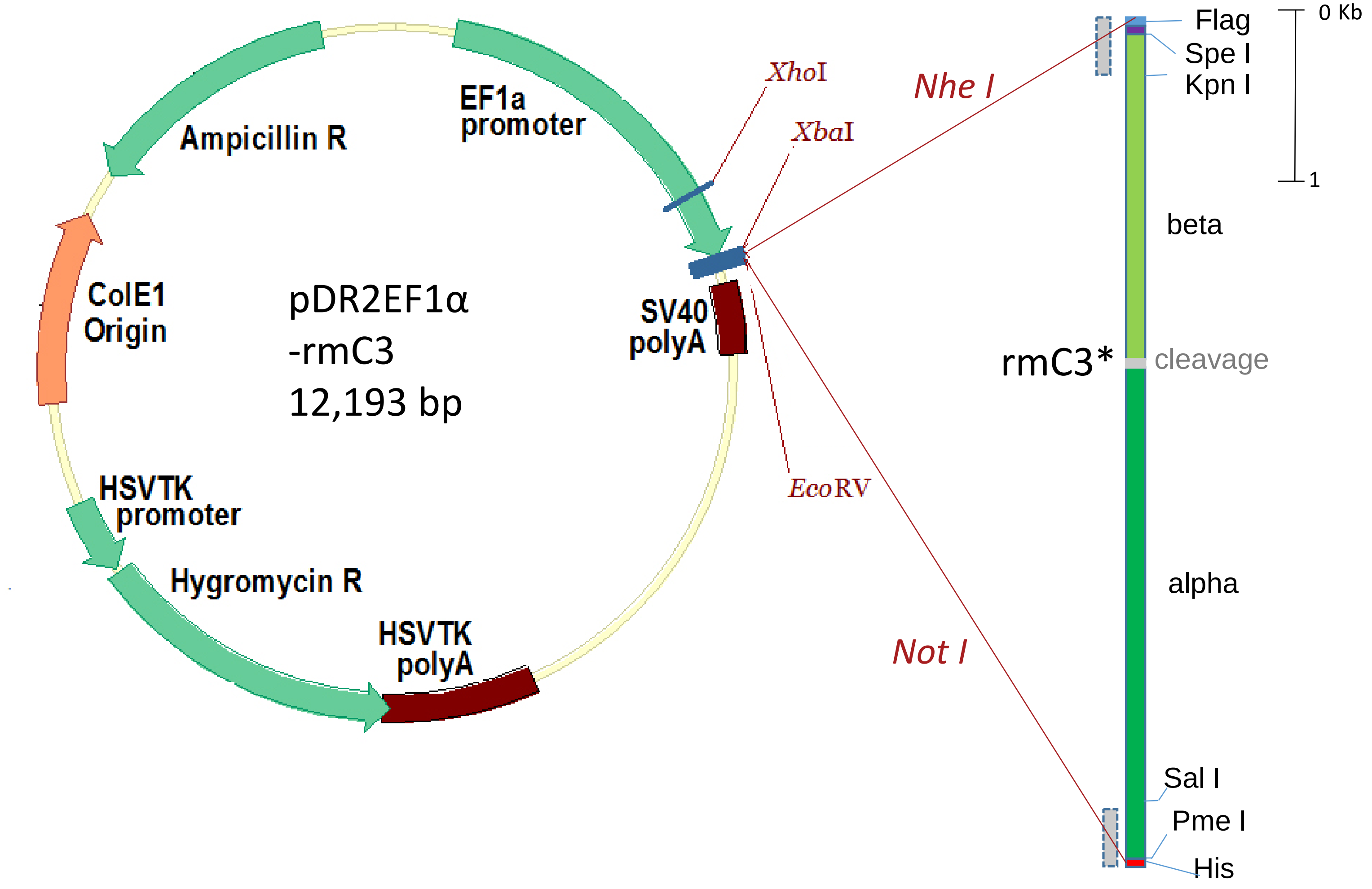

Diagram of the genetic organisation of the recombinant mouse (rm)C3 cDNA in the eukaryotic expression vector pDR2ΔEF1α. Restriction digest sites and purification tags are indicated. Mouse C3 was restriction digest cloned from a pCMV-Sport6 vector containing image clone 5124713* (with NCBI accession number = BC043338.1; Source Bioscience) using Kpn I and Sal I restriction sites. His = refers to a 7x histidine repeat and Flag to the DYKDDDDK amino acid sequence engineered into sequence as outlined in the supplementary methods (a guide to the synthetic DNA generated to allow construction of the tagged rmC3 is illustrated by grey dashed boxes). Primers used to confirm the integrity of the plasmid are fully outlined in supplemental Table 3.

Supplementary Figure 4.

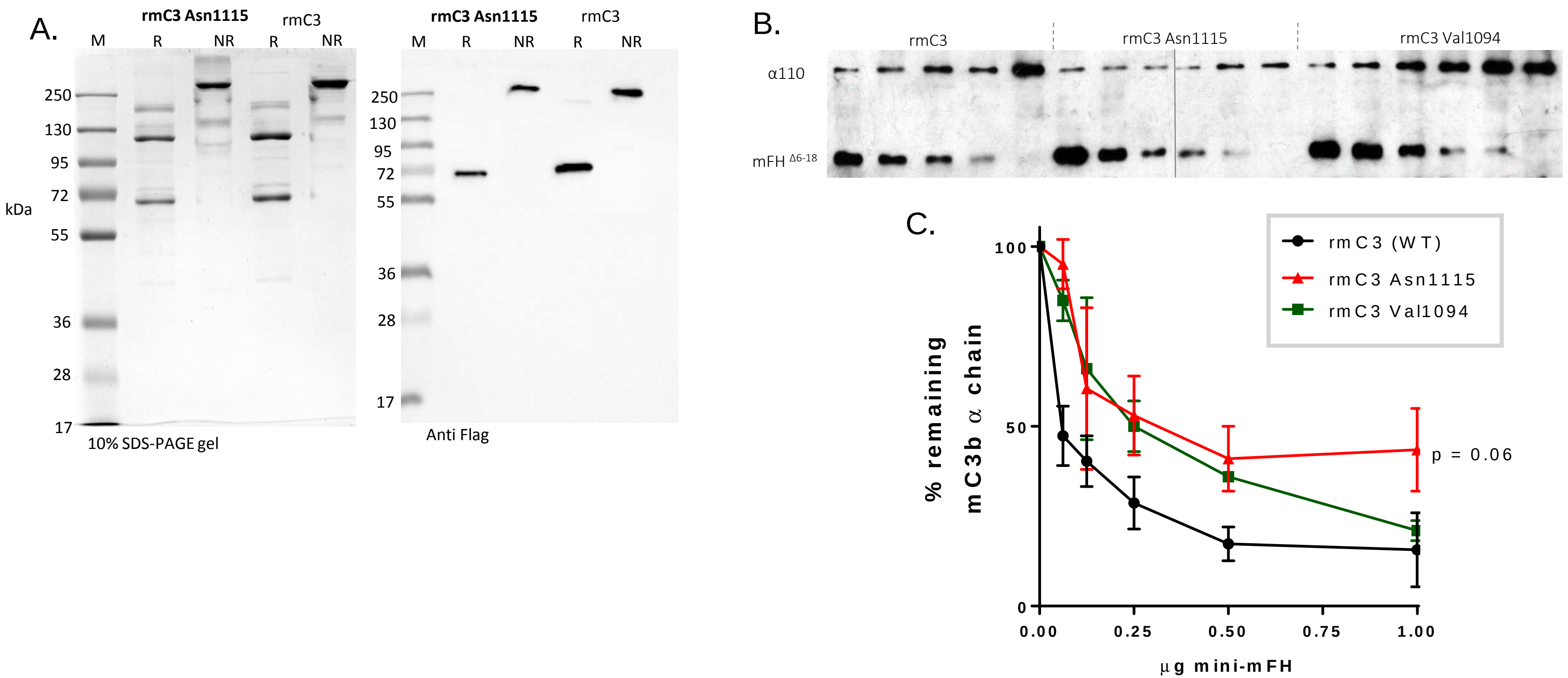

Analysis of recombinant mouse C3. (A) Recombinant wild type mouse C3 (rmC3) and recombinant mutant protein (rmC3^{D1115N}) were purified from CHO cell supernatant using FLAG^{Tm} tag affinity chromatography and elution using 0.1M glycine (immediately neutralised with 1M Tris pH 8). A 10% SDS-PAGE with loaded with 2µg of rmC3^{D1115N} or rmC3 non-reduced (NR) or reduced (R) to confirm purity. Western blot analysis (0.2µg/ml loaded) using monoclonal anti-FLAG antibody M2 (Sigma, UK) confirms the presence of intact C3 and C3β chain under NR and R conditions, respectively. Pre-stained markers were included (M; Frementas, UK) and sizes are indicated on the left of the SDS-PAGE gel. **(B)** 5 µg of affinity purified recombinant mouse c3 (rmC3) and mutants Val1094, Asn1115 (Met +1) were incubated with decreasing amounts of mini-mFH (minimal mouse FH, SCR1-5 linked to 18-20) and serum derived mouse FI for 30 minutes at 37°C. Reduced samples (1/10 diluted) were applied to 10% page, western blotted and exposed to an anti-his tag antibody (Penta-His 1/1000, Qiagen) followed by 1/500 SA-HRPO (representative of 2 experiments). Dashed line indicates the division between two gels/westerns that were run back to back.

(C) Densitometry analysis of intact alpha chain, standardised to Beta chain load, as determined by Image Studio v5.2. N = 2 independent experiments, mean +/- SEM. Wilcoxon’s rank sum was used to establish significance of difference between rmC3 and rmC3 Asn1115.

Supplementary Figure 5.

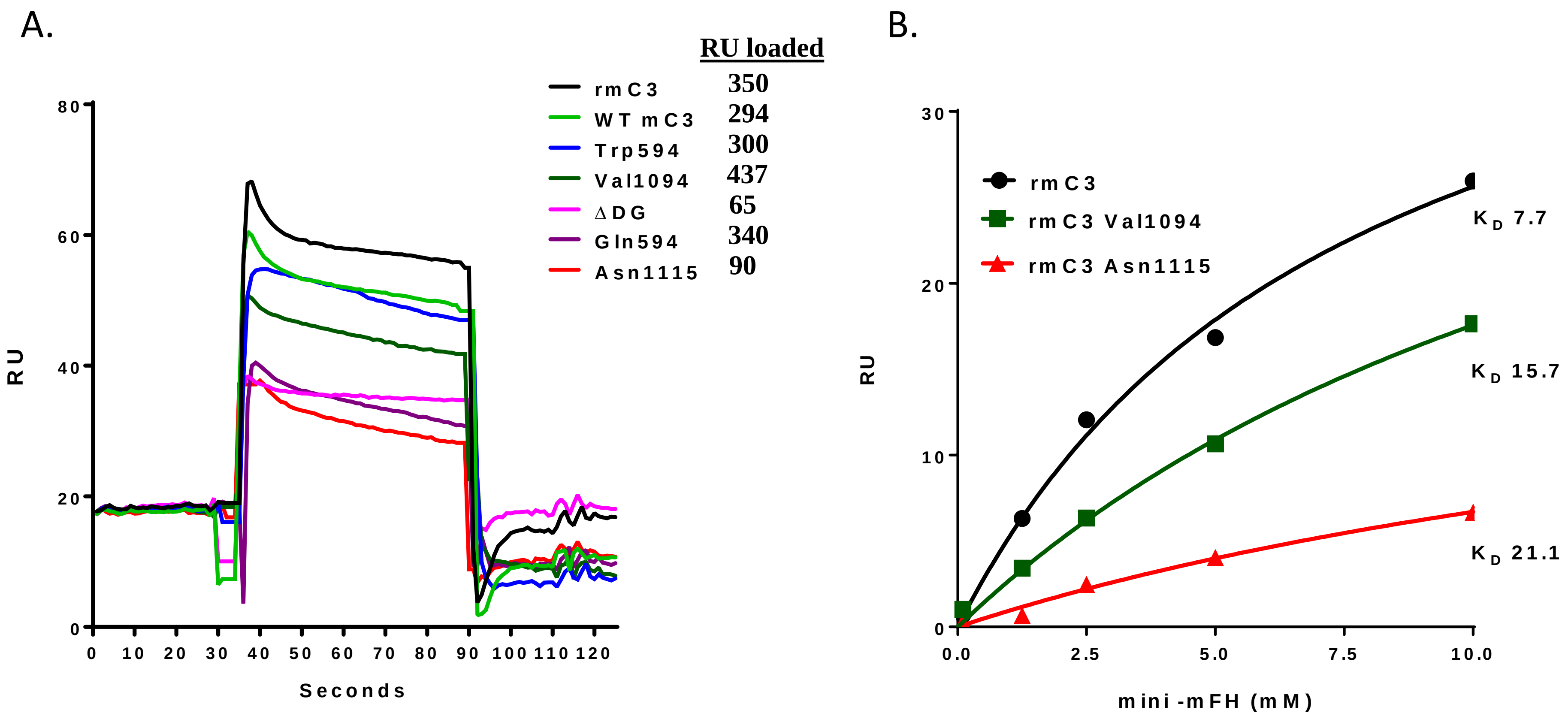

Analysis of recombinant mouse C3 using surface plasmon resonance (BIAcore).

A). Zeroed line graph of response units (RU) interaction of 20µM mini-mFH (mouse FH SCR1-5-18-20) binding to methylamine treated wild type, recombinant wild type or mutant proteins (Met + 1) C3 pre-captured onto a CM5 chip coated with 1000 RU of monoclonal antibody C3d11 (kind gift Prof M Holers, UCD, Denver, USA). A target of ~300 RU of each mouse protein was achieved (with the exception of ΔDG and Asp1115, as indicated) and given 300 seconds to stabilise. Mouse FH bound rmC3 more readily than WT mC3. Trp594 and Val1094 were comparable to WT C3, while Gln594, ΔDG and Asn1115 demonstrated the lowest binding capacity. Representative of 2 replicates. Data exported and analysed in Graphpad v7.

B). Increasing concentrations of mini-mFH were flowed over a CM5 chip pre-coated as above and the response (in RU) determined. K_D value were calculated by non-linear regression curve fit using Graphpad v7.

Supplementary Figure 6.

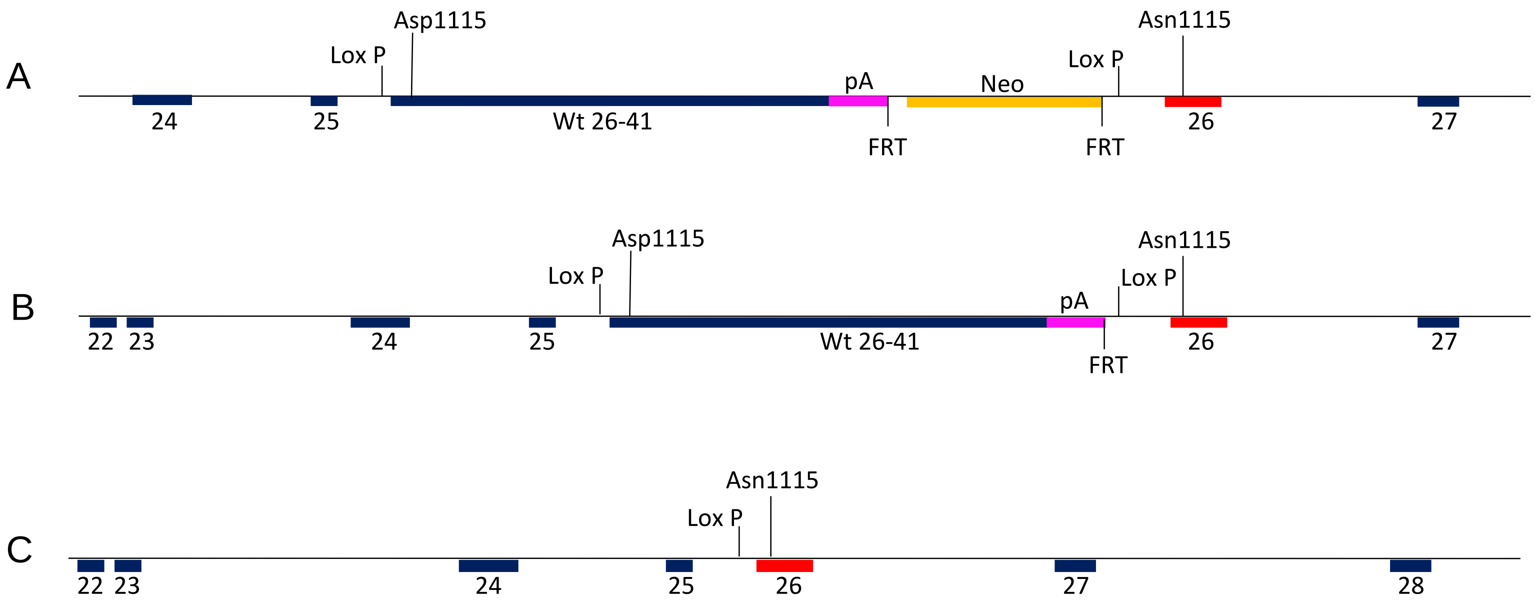

Making C3^{D1115N} conditional and constitutive KI mice. (A) Map of genetic organisation of the conditional knock-in (ConKI) construct used to transfect ES cells; wt26-41 sequence was obtained from source bioscience, image clone 5134713 **(B)** map showing genetic organisation after cross with Flpe mice (C3-ConKI-Flp) **(C)** Map showing genetic organisation of C3 KI mice after cross breed with OzCre mouse. Exons: Blue boxes; Neo: neomycin cassette for selection of ES cells; FRT; recognition sequence for Flp recombinase mediated neo removal; LoxP: recognition sequence for cre recombinase mediated wt mouse *C3* cDNA

Supplemental Figure 7.

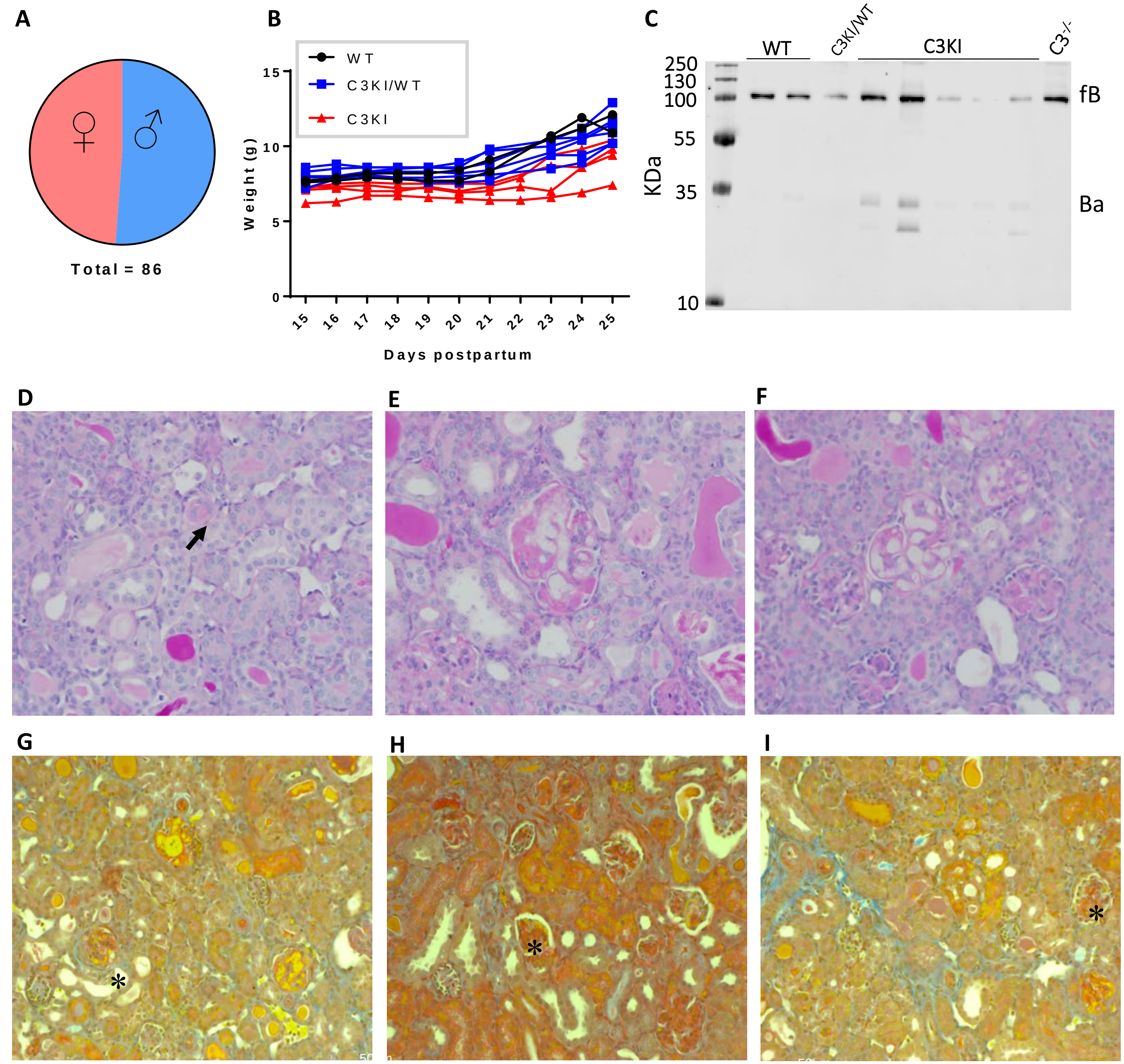

Additional analysis of the C3KI mice.
(A) No sex differences in the number of C3KI bred**.** **(B)** C3KI (red triangles) show a failure to thrive in comparison to their litter mate controls. **(C)** Western blot of murine plasma (freshly collected into EDTA and run under non-reduced conditions). A polyclonal goat anti-human factor B (1/1000, Comptech, Tyler, Tx) and donkey anti-goat HRPO (1/5000, Jackson Labs, Stratech, UK) was used to visualise murine factor B; C3 knockout mouse plasma was used as control for factor B breakdown. **(D-F)** PAS stain of C3KI mice showing an intravascular thrombus (arrow) (28 days old), representative images of chronic TMA in the C3KI mice. **(G-I)** MSB stains from C3KI mice showing a TMA with fibrin deposition within the glomeruli (asterix).

Supplemental Figure 8.

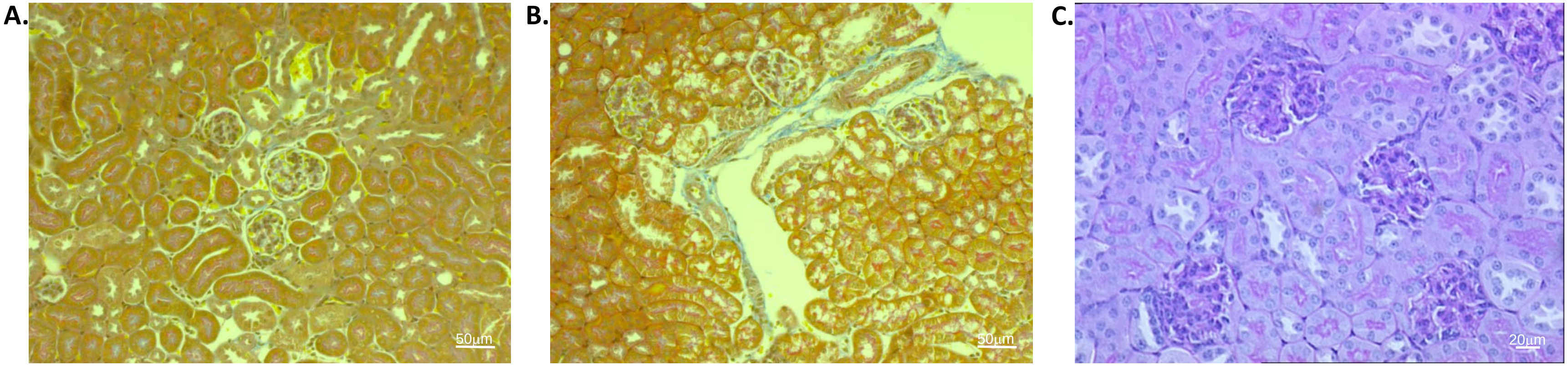

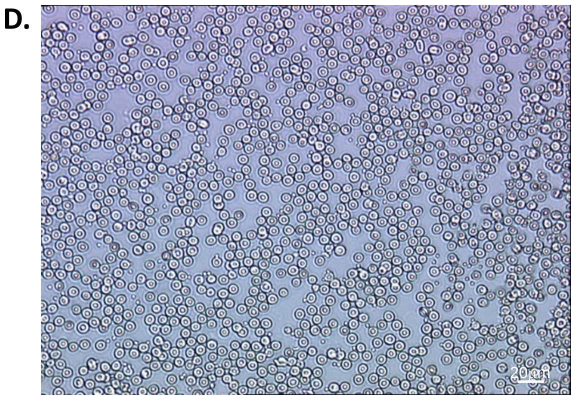

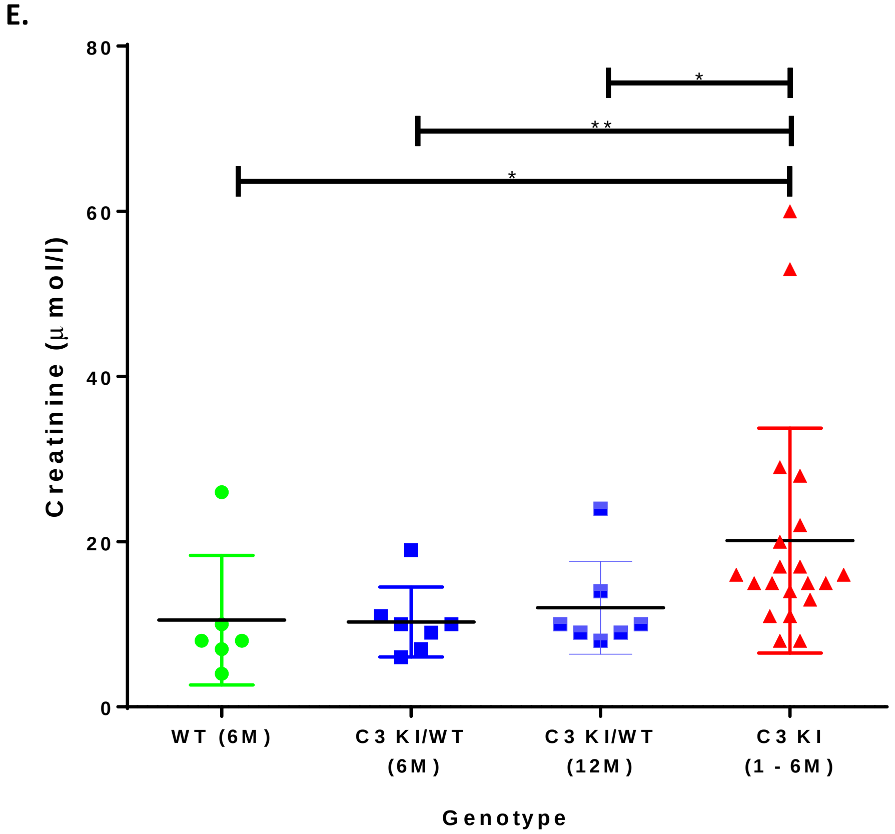

The C3KI/WT mouse (A, B) MSB stains from 4 week old C3KI/WT showing no evidence of fibrin deposition within the glomeruli. **C)** PAS stained section from 9 week old C3KI/WT showing normal glomeruli **(D)** Blood film from C3KI/WT showing no evidence of a MAHA **(E)** Creatinine measurements from 6 month (6M) old WT and C3KI/WT, 12 month old C3KI/WT mice compared to C3KI homozygotes aged 1 to 6 months (2 mice reached 6 months of age). Unpaired T-Test with Welch's correction. *P,<0.05; **p,<0.01.

Supplemental Figure 9.

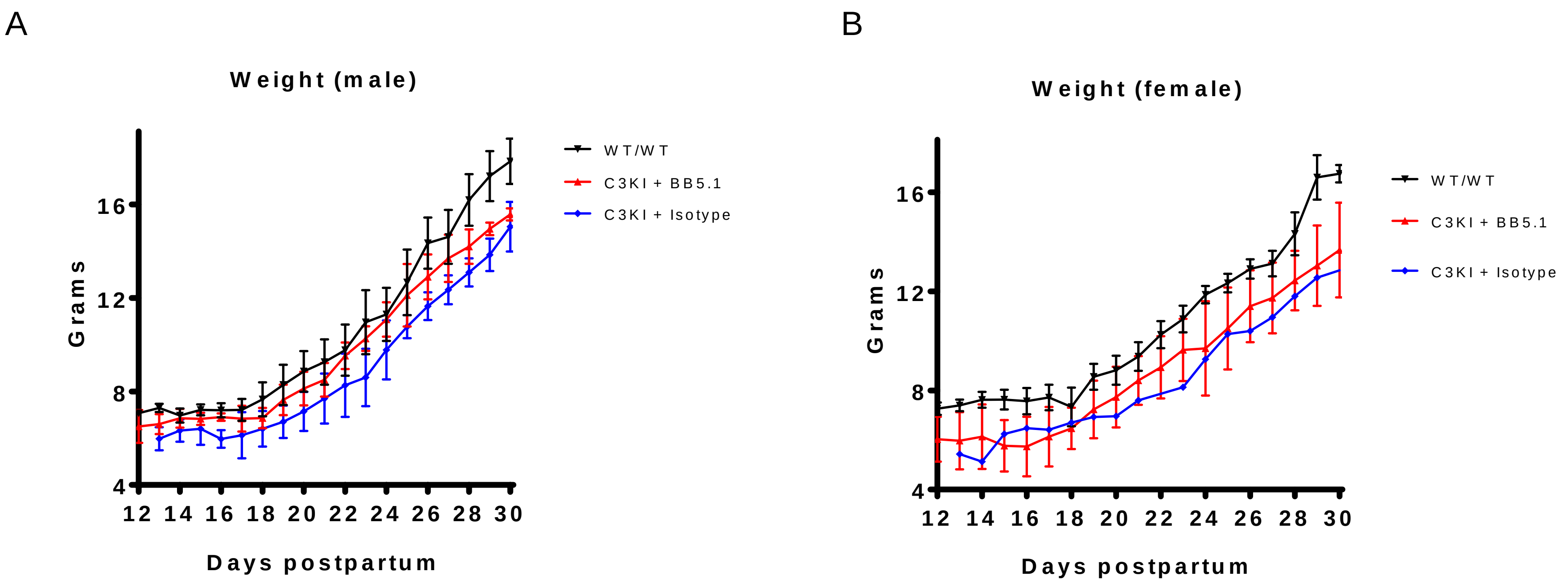

Weight Trajectory of C3KI mice.
(A) Weight gains of male C3KI mice in both treatment arms in comparison to wild type. **(B)** Weight gains of female C3KI mice in both treatment arms in comparison to wild type. Mice were weighted daily from post-partum day 12. N = 6 mice on average for each group.

Supplemental Figure 10.

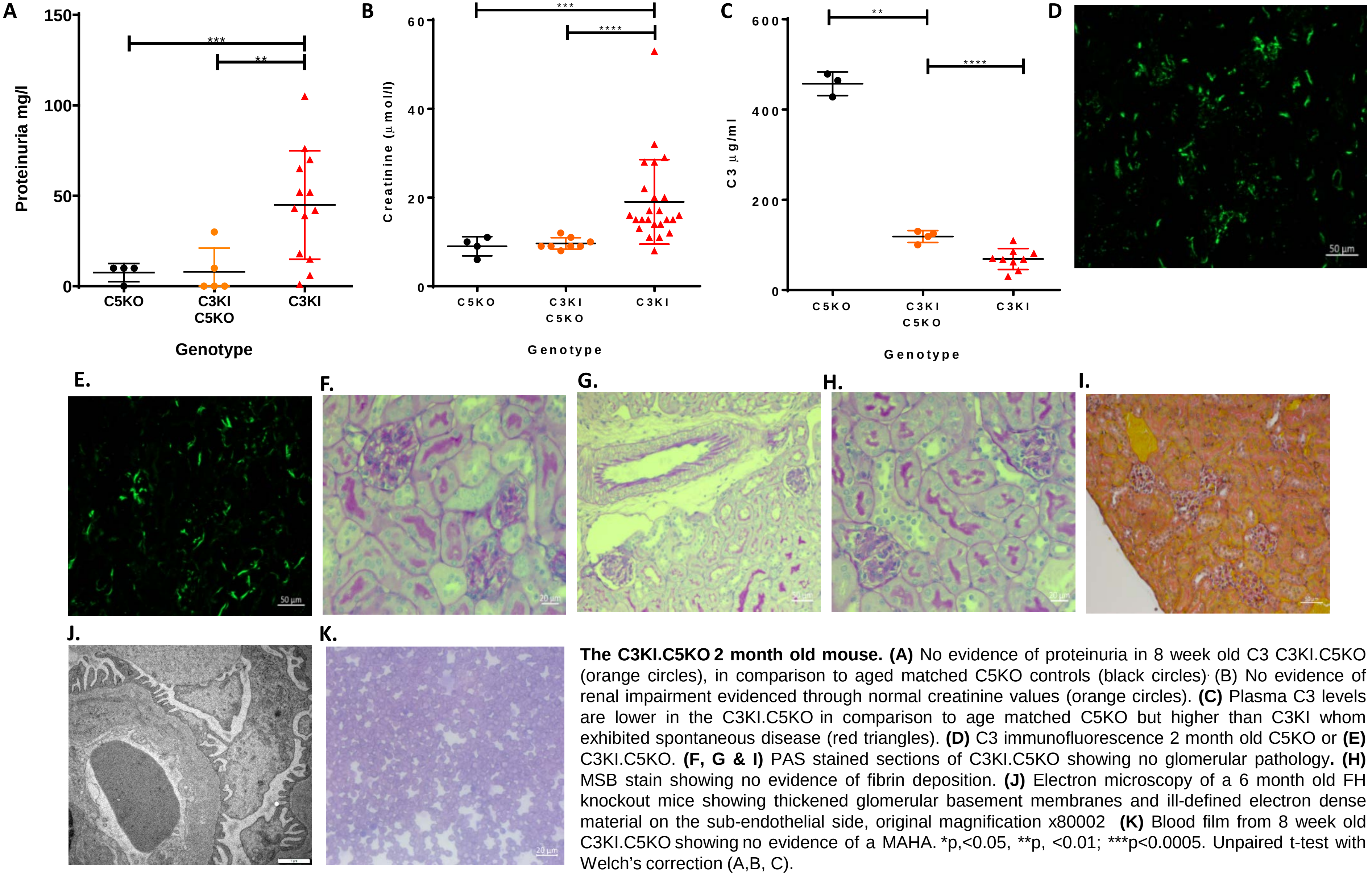

The C3KI.C5KO 2 month old mouse. (A) No evidence of proteinuria in 8 week old C3 C3KI.C5KO (orange circles), in comparison to aged matched C5KO controls (black circles). (B) No evidence of renal impairment evidenced through normal creatinine values (orange circles). **(C)** Plasma C3 levels are lower in the C3KI.C5KO in comparison to age matched C5KO but higher than C3KI whom exhibited spontaneous disease (red triangles). **(D)** C3 immunofluorescence 2 month old C5KO or **(E)** C3KI.C5KO. **(F, G & I)** PAS stained sections of C3KI.C5KO showing no glomerular pathology**. (H)** MSB stain showing no evidence of fibrin deposition. **(J)** Electron microscopy of a 6 month old FH knockout mice showing thickened glomerular basement membranes and ill-defined electron dense material on the sub-endothelial side, original magnification x80002 **(K)** Blood film from 8 week old C3KI.C5KO showing no evidence of a MAHA. *p,<0.05, **p, <0.01; ***p<0.0005. Unpaired t-test with Welch's correction (A,B, C).

Supplemental Table 1: Forward SDM oligonucleotide sequences used in the study

Name	Sequence (5’ to 3’)
G103R	GGAATTCAACTCAGATAAGGAGCGGCACAAGTACGTGACAGTGG
T163K	GCCCGTGGGCAAGAAAGTCGTCATCCTC
T163R	GCCCGTGGGCAAGCGAGTCGTCATCCTC
P370S	TTCAAGCCAGCCATGCCCTTTGACCTCATGGTGTT
R594Q	GGAAACCAGGGGGCCCAGGTGGGGCTAGTGGC
R594W	GGAAACCAGGGGGCCTGGGTGGGGCTAGTGGC
R735W	GCTGCAACCACATCACCAAGCTGTGGGAACAACACAG
(923)ΔDG	GTCTTCAATCACTTCATCA^GTGTCAAGAAGACACTGAAG
A1094V	CTCTCTAGCTGCCAACCTCATCGTCATCGACTCTCACGTCC
D1115N	GGAGAAACAGAAGCCGAATGGTGTCTTTCAGGAGG
Q1161K	CCAGGGACATCTGTGAGGGGAAGGTCAATAGCCTTCCTGGG
H1462D	CCTGACCTTCAAAGTTGACCAGTACTTTAATGTGGGACTTATCCAGCCC

Notes: Reverse complement sequence used for reverse SDM oligo. Altered bases, compared with consensus sequence are underlined and ^ indicates the deletion of sequence, as appropriate. Numbering from Met+1 according to consensus alignment in supplementary Figure 1A.

Supplemental Table 2: Multiple cloning site 3 oligonucleotides

Name	Sequence (5’ to 3’)
nMCS-3 F	CTAGAGCTAGCTGATCACTAGTCGACCCGGGATCCGGTACCAAGCTTGC GGCCGCATCGAT
nMCS-3 R	ATCGATGCGGCCGCAAGCTTGGTACCGGATCCCGGGTCGACTAGTGATC AGCTAGCT

Supplemental Table 3: PCR oligonucleotides

Name	Sequence (5’ to 3’)
mC3 F1	GTGGGTGGATGTGAAGGATTCCTG
mC3 F2a	CCCAGCAGCTGGCCTTCAAACAGCCC
mC3 F3	CAAAGCCTTCTCCAACAAGAACACCC
mC3 F4	ATGGGACCAGCTTCAGGGTCCCAGCT
mC3 F5	GAAGGAATACGTGCTGCCCAG
mC3 F6	CAACCACATCACCAAGCTGCG
pDEF-NF	CTCAAGCCTCAGACAGTGGTT
FLAG Tag	GATTACAAGGATGACGATGACAAG
PME I/ Thrombin cleavage/ HIS Tag	AGTTTAAACCTAGTTCCTCGTGGATCA CACCATCACCACCATCACCAC
mFH18 - BF	GGGATATGGATCCAAAAACCAAAGTG CCGA
mFH1- XSF	CACTCTAGAGTCGACCCACGCGTCCG GAGAGG

Name	Sequence (5’ to 3’)
mC3 R1	CAACTCTTCTATGGTCCACAACCAGC
mC3 R2	CATCAGGGCCAGGGCATACCCAGC
mC3 R3	AGCAGTTCTCTTCAGCACACCGGCAC
mC3 R4	GGATGACGACTGTCTTGCCCACGGGC
mC3 R5	TGTTTCTGGTACTTCTGATCCTGGCATTCT
mC3 R6	GGGACAACCATAAACCACCATAGATTC
mC3 R7	GTTCTTCGCACTGTTTCTGGTACTTCTGAT
mC3 R8	CCACAGCCACTAGCCCCACTC
mC3 R9	GTGGGTGGATGTGAAGGATTCCTG
mC3 R10	CATGTTCTGTTCCCCACAGCC
pDEF- NR	CTGAGGCTTGAGAATGAACC
mFH5 - BR	AGGGATCCAGCCAGTGGGTGTACA
mFH20-His-NHR	GCCGCTAGCTTAGTGATGGTGGTGATGGTGGT GTACACAAGTGGGATAATT

Figure 2a – uncropped, data used

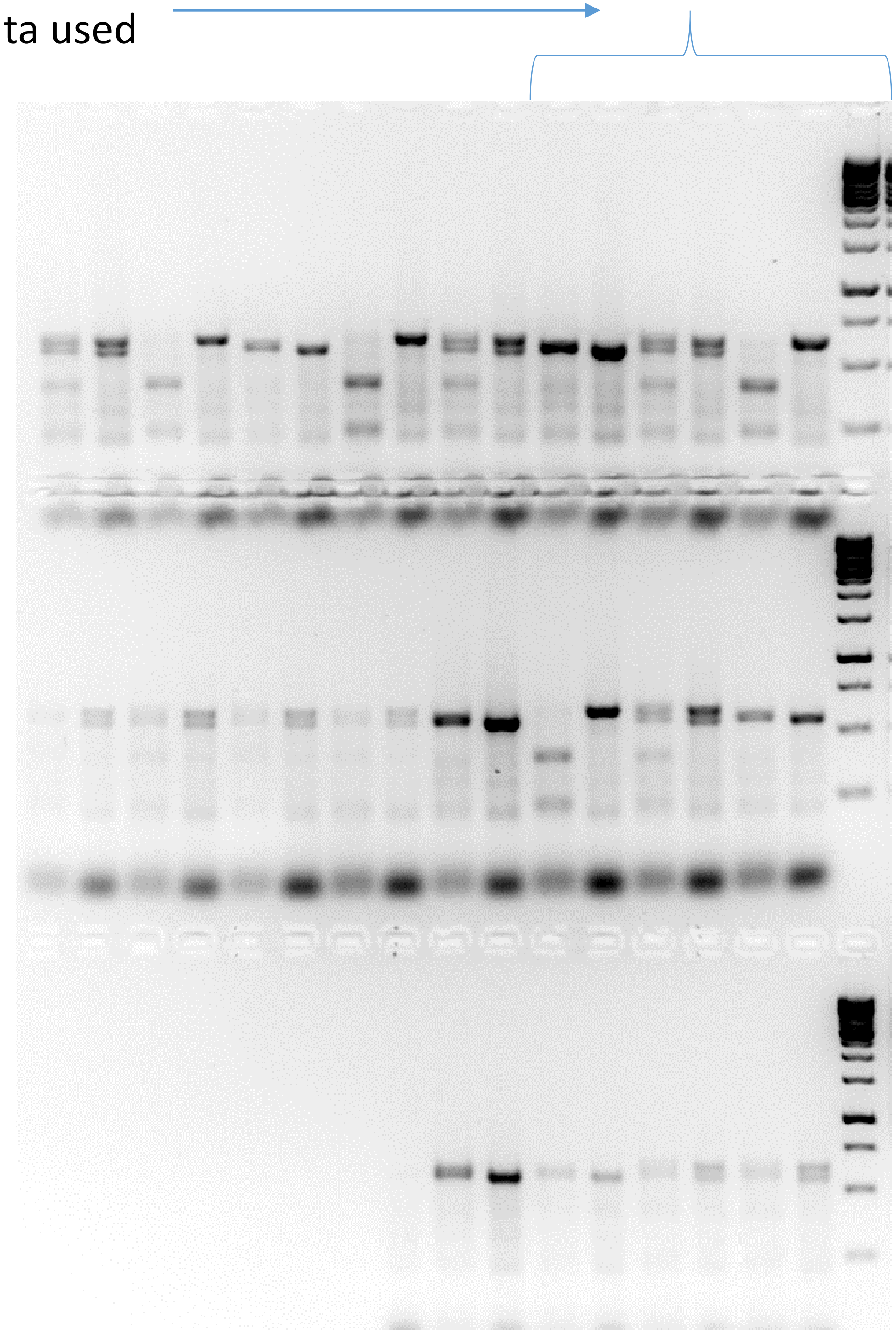

Figure 2C – uncropped, all data used

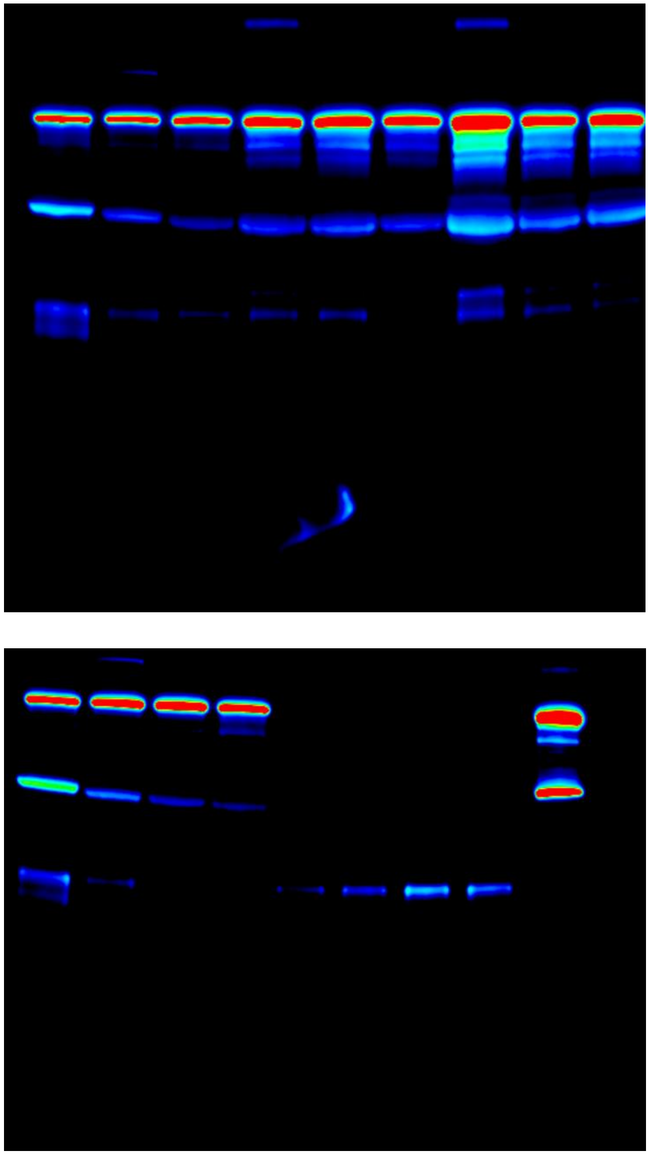

Figure 6E – uncropped, all data used

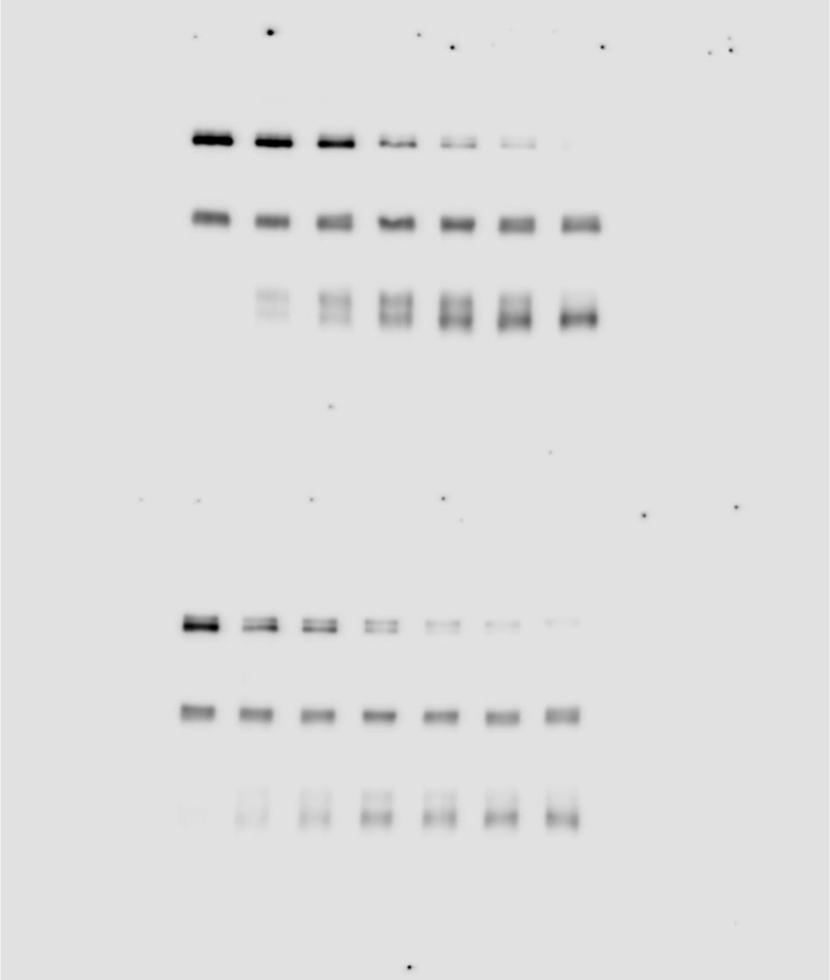

Supplemental Figure 4a – uncropped,

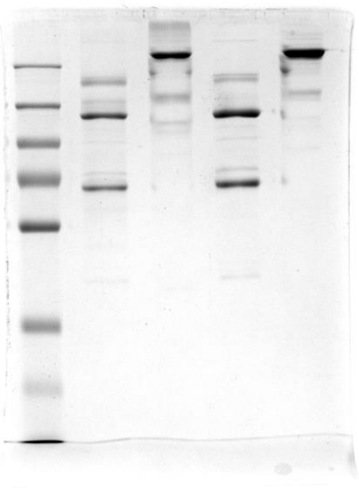

Bottom of gel cropped
in image, all data used

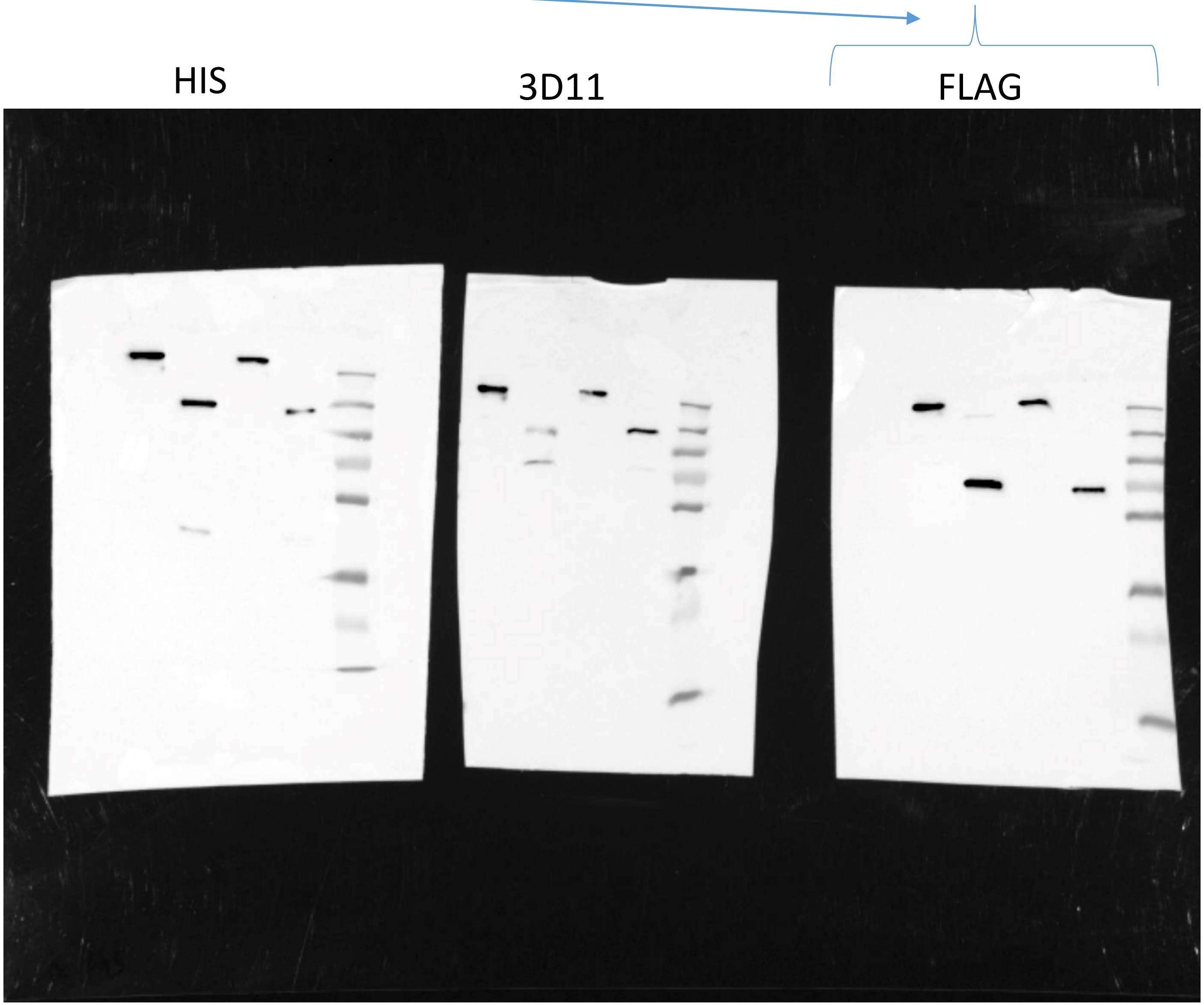

Supplemental Figure 4b – uncropped,

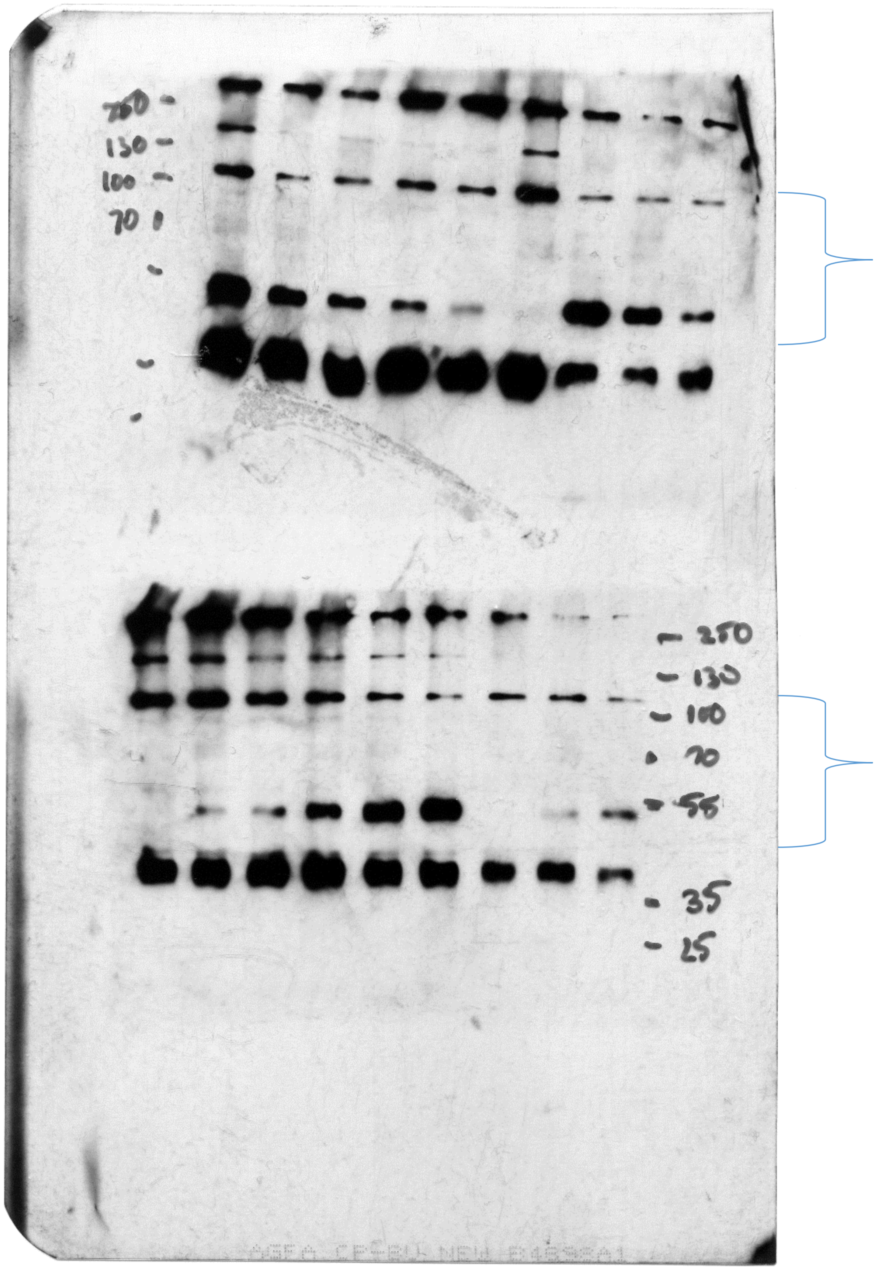

Supplement: Supplemental data [file jci-129-99296-s152.pdf]
